# Insect fungal pathogens secrete a cell wall-associated glucanase that acts to help avoid recognition by the host immune system

**DOI:** 10.1371/journal.ppat.1011578

**Published:** 2023-08-09

**Authors:** Huifang Wang, Zhuoyue Lu, Nemat O. Keyhani, Juan Deng, Xin Zhao, Shuaishuai Huang, Zhibing Luo, Kai Jin, Yongjun Zhang

**Affiliations:** 1 Key Laboratory of Agricultural Biosafety and Green Production of Upper Yangtze River (Ministry of Education), College of Plant Protection, Southwest University, Chongqing, People’s Republic of China; 2 Key Laboratory of Entomology and Pest Control Engineering, Academy of Agricultural Sciences, Southwest University, Chongqing, People’s Republic of China; 3 Department of Biological Sciences, University of Illinois, Chicago, Illinois, United States of America; 4 Genetic Engineering Research Center, School of Life Sciences, Chongqing University, Chongqing, People’s Republic of China; Ohio State University, UNITED STATES

## Abstract

Fungal insect pathogens have evolved diverse mechanisms to evade host immune recognition and defense responses. However, identification of fungal factors involved in host immune evasion during cuticular penetration and subsequent hemocoel colonization remains limited. Here, we report that the entomopathogenic fungus *Beauveria bassiana* expresses an endo-β-1,3-glucanase (BbEng1) that functions in helping cells evade insect immune recognition/ responses. *BbEng1* was specifically expressed during infection, in response to host cuticle and hemolymph, and in the presence of osmotic or oxidative stress. BbEng1 was localized to the fungal cell surface/ cell wall, where it acts to remodel the cell wall pathogen associated molecular patterns (PAMPs) that can trigger host defenses, thus facilitating fungal cell evasion of host immune defenses. BbEng1 was secreted where it could bind to fungal cells. Cell wall β-1,3-glucan levels were unchanged in *ΔBbEng1* cells derived from *in vitro* growth media, but was elevated in hyphal bodies, whereas glucan levels were reduced in most cell types derived from the *BbEng1* overexpressing strain (*BbEng1*^*OE*^). The *BbEng1*^*OE*^ strain proliferated more rapidly in the host hemocoel and displayed higher virulence as compared to the wild type parent. Overexpression of their respective *Eng1* homologs or of *BbEng1* in the insect fungal pathogens, *Metarhizium robertsii* and *M*. *acridum* also resulted in increased virulence. Our data support a mechanism by which BbEng1 helps the fungal pathogen to evade host immune surveillance by decreasing cell wall glucan PAMPs, promoting successful fungal mycosis.

## Author summary

Fungal pathogens infect insects via penetration through the host cuticle and proliferation within the hemocoel. Important aspects of how the fungus evades host immune defenses remain poorly understood. Here, we report that a secreted / cell-wall associated endo-β*-*1,3-glucanase (BbEng1) expressed by *Beuaveria bassiana* during host infection acts to help the pathogen evade host immune responses by reducing levels of cell wall glucans that act as pathogen associated molecular patterns (PAMPs) recognized by host defenses that act to induce immune responses. Loss of BbEng1 did not alter cell wall glucan levels in cells derived from *in vitro* growth media, but elevated in hyphal bodies, whereas overexpression of *BbEng1* decreased cell wall glucan contents, with the latter resulting in increased virulence. BbEng1 homologs were also shown to contribute to virulence in the insect fungal pathogens *Metarhizium robertsii* and *M*. *acridum*. These findings uncover a critical molecular determinant employed by fungal pathogens to help evade host recognition that would otherwise induce subsequent immune defense responses.

## Introduction

Microbial pathogens and their hosts engage in counter-selecting adaptations that define important elements in their co-evolutionary “arms race” [1]. Fungal pathogens have evolved the ability to invade and ultimately consume their hosts. In response, hosts have evolved defense systems that include immune pathways that function to suppress and kill invading parasites. Fungal parasites develop strategies to overcome host defense barriers, which can, in turn, select for additional host defenses that the pathogen must then overcome [1–3]. Insect pathogenic fungi have evolved a wide range of adaptations to overcome the formidable defenses of hosts. These adaptations include both the production of (insect) toxins and the ability to detoxify host microbe-targeting toxins and antimicrobial compounds, the production of a battery of enzymes capable of degrading host structures and tissues, and mechanisms of evading host immune responses that has even included the incorporation (via horizontal gene-transfer) of insect genes into the fungal genome that increases the virulence of the fungus [4–6].

Insects are considered to lack the canonical acquired immune pathways seen in vertebrates but have evolved innate immune systems that include cellular and humoral pathways that function to block microbial infections [3]. Entomopathogenic fungi typically infect host insects via cuticular penetration, which is initiated after attachment of the conidial (spores) to the host epicuticle, followed by germination and formation of infective structures, such as appressoria or penetration pegs, that function to breach the cuticle using a combination of mechanical pressure and the actions of secreted cuticle-degrading enzymes [7]. Similar to some plant and animal fungal pathogens, once penetrating invasive hyphae reach the hemocoel, they undergo a dimorphic transition, elaborate free floating yeast-like cells, called *in vivo* blastospores or hyphal bodies, that are able to evade immune responses [8,9]. During infection, fungal tactics used to evade insect immune defense response are diverse, and include a variety of means to evade, inhibit, detoxify, and/or eliminate host immune responses [3,10]. Mechanism used by members of *Metarhizium* genera of insect pathogens includes the formation of a collagenous protective coat on the hyphal bodies that acts to mask pathogen associated molecular patterns (PAMPs) that are recognized by the host immune systems in order to activate antimicrobial defenses [11]. However, this mechanism does not appear to be present in the related *Beauveria* genera of insect fungal pathogens. *Beauveria bassiana* secretes a laccase that inhibits the production of host reactive oxygen species (ROS) as well as oxidizes various (potentially toxic) insect phenolic substrates [12]. In addition, entomopathogenic fungi produce an array of metabolites, toxins, effectors, and other small soluble molecules that deactivate or repress host immune cascades [13–16] and/ or help overcome insect-generated cytotoxic intermediates [4,12]. Various entomopathogenic fungi have also evolved species specific offensive capabilities, e.g., the secreted catalase-peroxidase produced by *M*. *acridum* that acts on the surface of the host during initial stages of infection [17].

One major host defense mechanism is the recognition of fungal cell wall β-1,3-glucans (that acts as a PAMP) to initiate host immune defenses via the well characterized Toll signaling pathway [18]. Within this context, the ability to mask and/or reduce cell wall glucan levels could be considered as a potential mechanism for reducing host immune recognition and subsequent activation. As a broad host range insect pathogen, *B*. *bassiana* is considered a more environmentally and human health friendly product as compared to chemical pesticides, and has been commercialized for use in the biological control of a wide range of arthropod pests [4,19,20]. Previously, we have shown that a secreted protein containing a conserved domain of the GH16 glucanase family was found in the cell surface and secreted protein pools of *B*. *bassiana* hyphal bodies during hemocoel infection of the silkworm *Bombyx mori* [21]. However, the function of the protein was not determined. Here, we have genetically and biochemically characterized this (endo-β*-*1,3-) glucanase (named BbEng1). Our data show that *BbEng1* is expressed in response to signals from insect cuticles and hemolymph as well under conditions of osmotic and oxidative stresses. BbEng1 was shown to localize to the fungal (hyphal body) cell wall/surface, decreasing cell wall β-1,3-glucans levels, thus presumably resulting in decreased host recognition and subsequent failure of the host to initiate aspects of its (antimicrobial) immune response. Overexpression of *BbEng1* resulted in greater levels of glucan reduction on fungal hyphal bodies and increased virulence as compared to the wild type parent. Orthologs of *BbEng1* in *Metarhizium* appear to perform a similar function, indicating this to be a general mechanism used by entomopathogenic fungi to help evade host immune recognition.

## Results

### BbEng1 is induced by insect-derived nutrients in *B*. *bassiana*

BbEng1 (BBA_04753) was previously isolated from the cell surface of *B*. *bassiana* insect hemocoel derived hyphal bodies as well as found (secreted) in the hemolymph from fungus infected silkworm larvae [12]. The protein consists of 588 amino acids, and features a putative N-terminal signal peptide (21 amino acids) and a putative conserved GH16 family glucanase domain (residues 22 to 343), which contains the catalytic motif (E-[ILV]-D-[IVAF]-[VILMF](0,1)-E) typically found in this family of proteins, and which includes two critical glutamic acids (Glu141 and Glu146) involved in catalysis as well as a conserved aspartic acid residue (Asp143) [22–24] (S1 Fig). In addition, BbEng1 contains 19 cysteine residues with the potential to make disulfide bridges. Phylogenetic analyses revealed broad distribution for BbEng1, that clustered to three distinct clades that mirrored host associations, *i*.*e*., into mammalian, plant and insect pathogens (S1 Fig).

**Fig 1:**
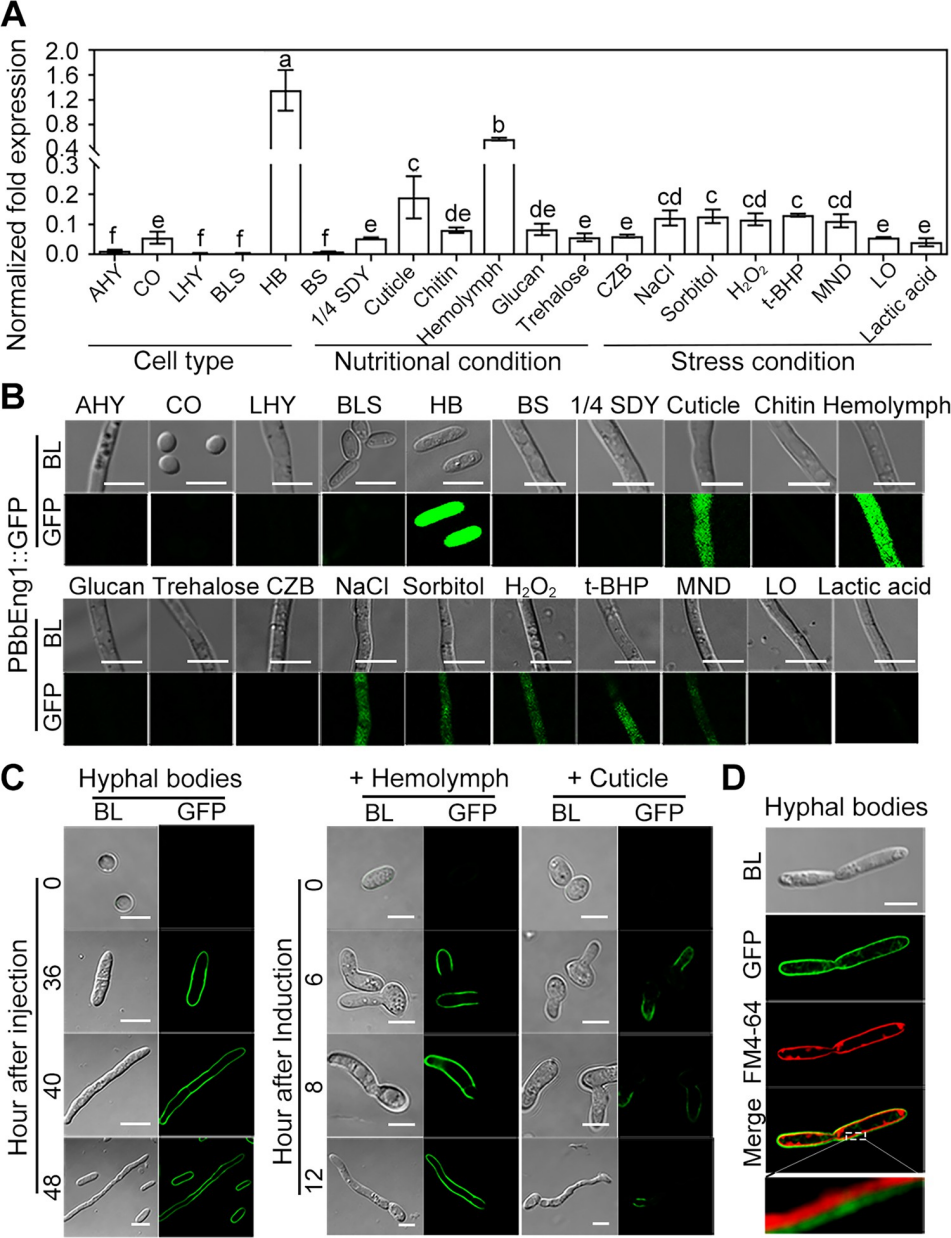
Gene and protein expression of BbEng1 in *B*. *bassiana*. **A.** Transcript levels of *BbEng1* in different morphological cells and cultures grown in different media and under stress conditions. AHY, aerial hyphae. CO, conidia. LHY, submerged hyphae. BLS, blastospores. HB, hyphal bodies (*in vivo* blastospores). BS, the basic salt broth. Cuticle and hemolymph, BS + 0.167 g / L silkworm cuticle or 5 mL / L hemolymph. Chitin, glucan or trehalose, used as sole carbon source in CZB as a substitute for sucrose at 2%. NaCl, sorbitol, H_2_O_2_, MND, t-BHP or lactic acid, CZB containing 0.5 M NaCl, 1.0 M sorbitol, 5.76 mM H_2_O_2_, 37 μM menadione or 0.78 mM tert-Butyl hydroperoxide or 2% (v/v) lactic acid. LO, low oxygen condition (with ~6% of initial O_2_ concentration). All fungal cells were cultured 6 h in different media and under different stress conditions. Error bars denote standard deviations (SDs) from three biological replicates. Different letters (a–f) indicate statistically significant differences (*P* < 0.01 in LSD test). **B.** Microscopic images (scale: 5 μm) of GFP fluorescence expressed in fungal cells of PBbEng1::GFP strain. *GFP* was driven by BbEng1 promoter. **C.** Microscopic images (scale: 5 μm) of GFP fluorescence in BbEng1::GFP hyphal bodies and cells cultured in insect-derived nutrients. Hyphal bodies were examined in hemolymph samples at the indicated time points after intra-hemocoel injection of conidia into *G*. *mellonella* larvae (left). Fungal cells grown in insect-derived nutrients (5 mL / L hemolymph or 0.167 g / L cuticle from silkworm) were collected at the indicated time points (right) of incubation. **D.** Microscopic images (scale: 5 μm) of GFP fluorescence and FM4-64 (stained cell membrane) in BbEng1::GFP hyphal bodies.

The expression pattern of *BbEng1* was examined via RT-qPCR and construction of a *BbEng1* promoter-GFP (PBbEng1::GFP) reporter strain (Fig 1A and 1B). These data showed that *BbEng1* was highly expressed in hemolymph derived hyphal bodies, but absent in *in vitro* cells, including conidia, submerged blastospores, and aerial and submerged hyphae grown under standard conditions in mycological media. *BbEng1* expression increased 20- to 60-fold in basal salt solution (BS) or Czapek-Dox broth (CZB) containing insect-derived extracts, including insect cuticle and hemolymph produced as detailed in the Methods section. *BbEng1* expression was also slightly induced by NaCl- and sorbitol-induced osmotic stress (~2.0-fold), and by oxidative stress (1.8–2.1-fold) as induced by addition of either tert-Butyl hydroperoxide, H_2_O_2_ or menadione to CZB. However, no obvious changes in *BbEng1* expression was seen under low oxygen stress (LO) or lactic acid-stress growth conditions. *BbEng1* expression was slightly (1.5–2 fold) elevated in cultures grown in 1/4 SDY and media supplemented with chitin, glucan or trehalose, however, no obvious differences in cells containing the GFP promoter fusion fluorescence construct (PBbEng1::GFP) in the same media.

### BbEng1 localizes to the cell wall/cell surface and is secreted during infection and in media containing insect cuticle or hemolymph

To examine localization of BbEng1, wild type *B*. *bassiana* was transformed to express a BbEng1::GFP fusion protein as detailed in the Methods section. No fluorescent signal was observed in conidia prior to injection into the insect hemocoel (0 h), with fluorescent signals appearing in hyphal bodies formed 36 h post-injection (Fig 1C). No fluorescence was seen in BbEng1::GFP cells at the initial period (at 0 h) after inoculation in insect cuticle or hemolymph-contained broth, but apparent in the fungal cells after 6 h of inoculation, particularly along/ within the cell wall and on the cell surface of germ tubes (Fig 1C). Moreover, signals could be clearly distinguished as occurring at the outer layer of the cellular membranes via counter staining with FM4-64 (Fig 1D).

**Fig 2:**
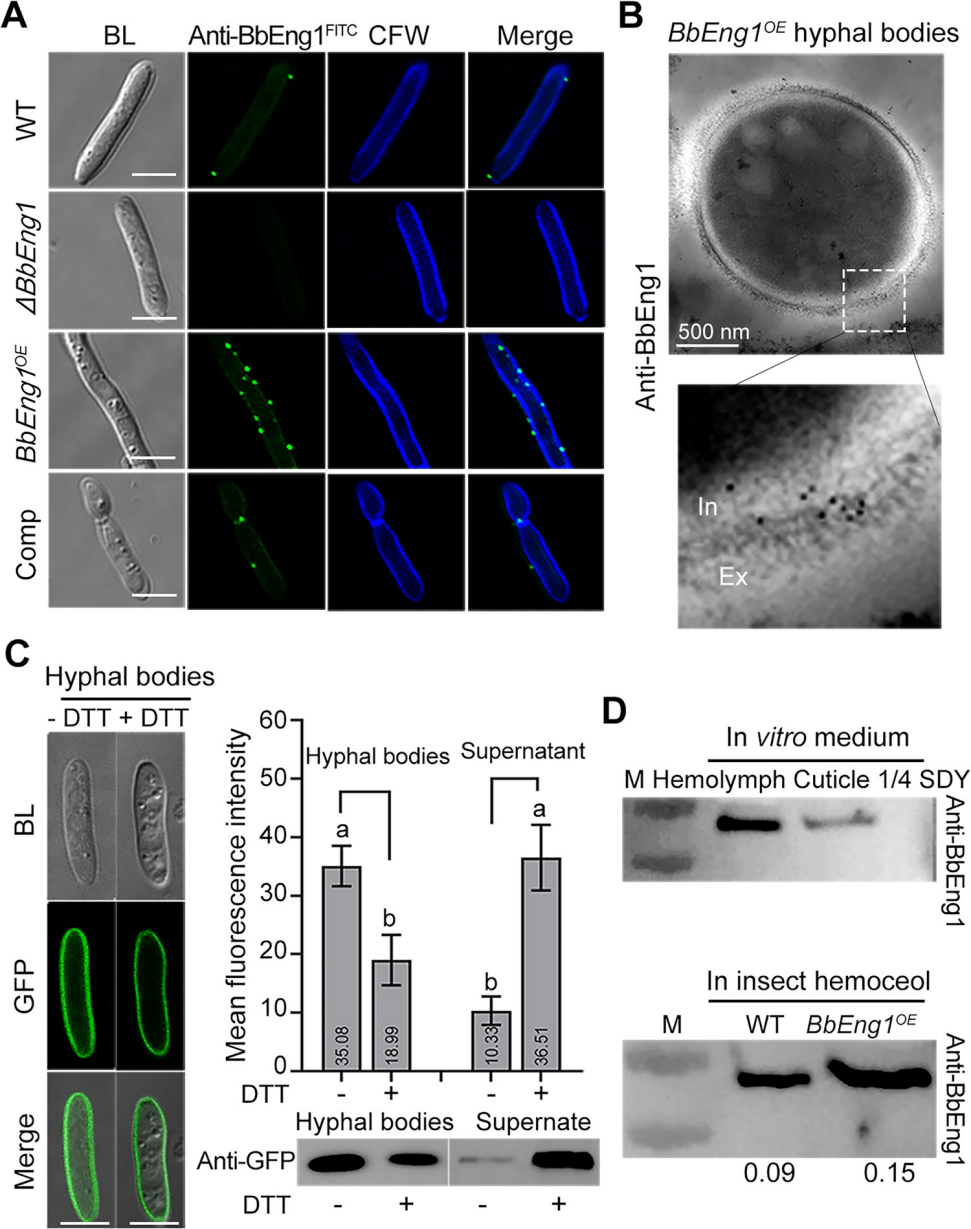
Distribution and secretion of BbEng1 in *B*. *bassiana*. **A.** Indirect immunofluorescence (IIF) images (scale: 5 μm) examining subcellular localization of BbEng1 in *B*. *bassiana* wild-type, *ΔBbEng1*, *BbEng1*^*OE*^ and complementation (Comp) hyphal bodies probed with anti-BbEng1 polyclonal antibody and FITC-conjugated-goat anti-rabbit IgG. Fungal cell wall was stained with CFW (calcofluor white). **B.** Immuno-transmission electron images of BbEng1 in hyphal bodies of *BbEng1*^*OE*^ probed with anti-BbEng1 polyclonal antibody and anti-Rabbit IgG (Whole molecule)-Gold. **C.** Microscopic images (scale: 5 μm) of BbEng1::GFP expressed in hyphal bodies after 12-h treatment with 2 mM dithiothreitol (DTT). Left, GFP fluorescence in fungal cells. Right, fluorescence density in the supernatant. The BbEng1::GFP cells and the supernatant were probed with anti-GFP antibody. Control (-), not treated with DTT. Error bars: SDs. Different letters indicate significant differences pairs (*P* < 0.01, *t*-test). **D.** Western blotting detection of secreted BbEng1 by anti-BbEng1 polyclonal antibody. Upper, wild-type incubated 12 h in insect-derived nutrient-contained broth (0.167 g / L silkworm cuticle; 5 mL / L silkworm hemolymph) and 1/4 SDY broth. Lower, insect hemolymph of larvae after 48-h injection with wild-type and *BbEng1*^*OE*^ conidia. The digital values denote the amount of BbEng1 secreted by the tested strains in the host hemolymph.

To characterize the function of the gene product, targeted gene knockout (*ΔBbEng1*), complementation (*ΔBbEng1*::*BbEng1*, Comp) and overexpression (*BbEng1*^*OE*^, ~200-fold upregulated in 1/4 SDY) strains were generated as detailed in the Methods section (S2 Fig). Using an indirect immunofluorescence (IFL) assay to probe cells with a polyclonal antibody generated towards BbEng1 (anti-BbEng1, see Methods) coupled to goat anti-rabbit IgG-FITC labeling, BbEng1 signals were seen as distributed as punctate spots on the outside edge of/ in the calcoflour white (CFW) counter stained cell (walls) in *B*. *bassiana* hyphal bodies (Fig 2A). The signal was significantly increased in *BbEng1*^*OE*^ hyphal bodies but was absent in *ΔBbEng1* cells (Fig 2A). Immuno-transmission electron micrographs revealed a localization of BbEng1 on the cell wall and its distribution on the cell surface of *BbEng1*^*OE*^ hyphal bodies (Fig 2B). Experiments using the BbEng1::GFP fusion construct bearing strain, indicated significantly decreased fluorescence signals (46%, *P* < 0.01) in hyphal bodies treated for 12 h with 2 mM dithiothreitol (DTT), with the fluorescence intensity in the surrounding solution (*i*.*e*., released into the media) increased by 2.5-fold in comparison to controls (not treated with DTT) (Fig 2C). To further measure the release of BbEng1::GFP from cell walls after DTT treatment, samples from the cell cultures treated without or with DTT were probed with anti-GFP antibody. The results confirmed “release” of BbEng1::GFP from cell wall fractions after treatment with DTT (Fig 2C). To determine whether BbEng1 was secreted in response to host cues, Western blots probed with an anti-BbEng1 antibody revealed the presence of secreted BbEng1 protein in *B*. *bassiana* grown in media supplemented with silkworm cuticle or hemolymph, but not when the fungus was grown in nutrient-rich 1/4 SDY. A secreted pool of BbEng1 was also detected *in vivo* from the hemolymph of *Galleria mellonella* 48 h after injection of wild type and *BbEng1*^*OE*^ conidia. Quantification of *in vivo* secreted signals indicated ~0.1 μg and 0.15 μg of BbEng1 per 100 μg of protein extract in hemolymph samples of wild type and *BbEng1*^*OE*^ infected insects, respectively (Fig 2D).

### Involvement of BbEng1 in cell wall remodeling

**Fig 3:**
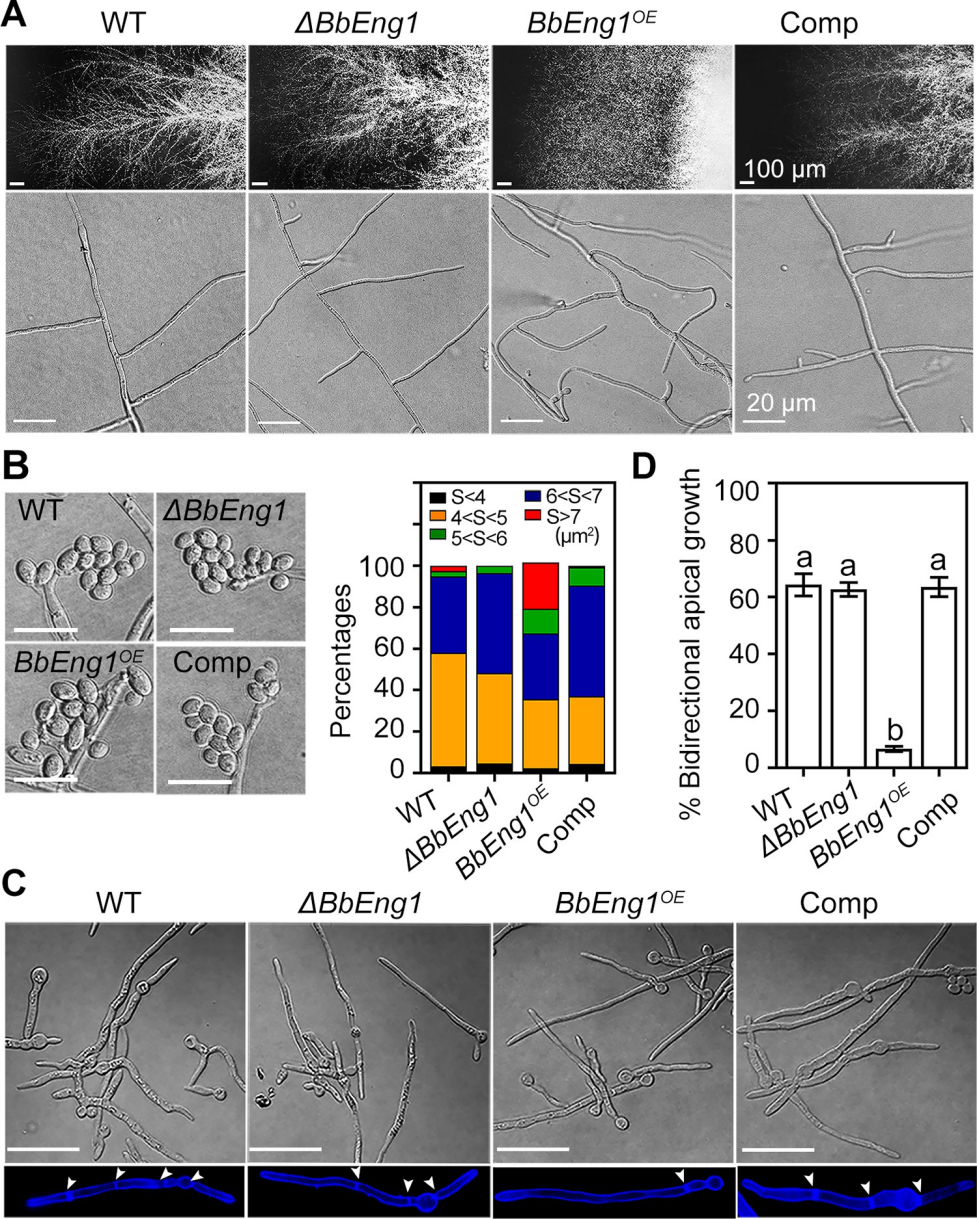
Morphological characterization and conidial germination of *B*. *bassiana* wild type and mutant strains. **A.** Colony edge and aerial hyphae morphology. **B.** Conidial morphology and size (scale: 10 μm). **C, D**. Morphology of conidial germination (scale: 20 μm) and ratio of bidirectional apical growth from conidia. Error bars in (D) denote SDs. Different letters indicate significant differences (*P* < 0.01 in LSD test).

The *ΔBbEng1*, *BbEng1*^*OE*^ and control strains were examined under different growth and developmental conditions. The *BbEng1*^*OE*^ strain showed a slight to moderate increase in colony growth (6–24%, *P* < 0.01) on nutrient-rich 1/4 SDAY, minimal medium CZA, and CZA modified with different carbon sources (S3 Fig), as well as moderate increases (20–65%, *P* < 0.01) in conidial production in various media (S3 Fig). *BbEng1*^*OE*^ conidia also more readily dissociated from conidiophores than the wild type strain (S3 Fig). *BbEng1*^*OE*^ colonies displayed denser mycelial edges than control strains (wild type and complemented strains), with an abnormal curled hyphal morphology apparent (Fig 3A). Ratios of polymorphic conidia (swollen and oval with section areas > 6 μm^2^) were significantly higher in the *BbEng1*^*OE*^ strain (34%, *P* < 0.01) than those of the wild-type conidia (5.1%) (Fig 3B). Examination of dimorphic transition showed that similar to conidiation, the *BbEng1*^*OE*^ strain also produced more blastospores as compared to the wild type strains (S4 Fig). *BbEng1*^*OE*^ conidia also germinated slightly more rapidly than wild type conidia, with a median germination time (GT_50_) shortened by 11–13% (*P* < 0.01) as compared to the control strains’ conidia (S3 Fig). In addition, all germinated *BbEng1*^*OE*^ conidia produced singular germ tubes (unidirectional hyphal growth) with fewer septum, contrasting with high incidences of bidirectional germination and hyphal growth (~63%) and higher septal density in the control strains (Fig 3C and 3D). No obvious differences were seen in colony growth, conidiation and germination as well as septation patterns between *ΔBbEng1* and control strains (Figs 3 and S3).

**Fig 4:**
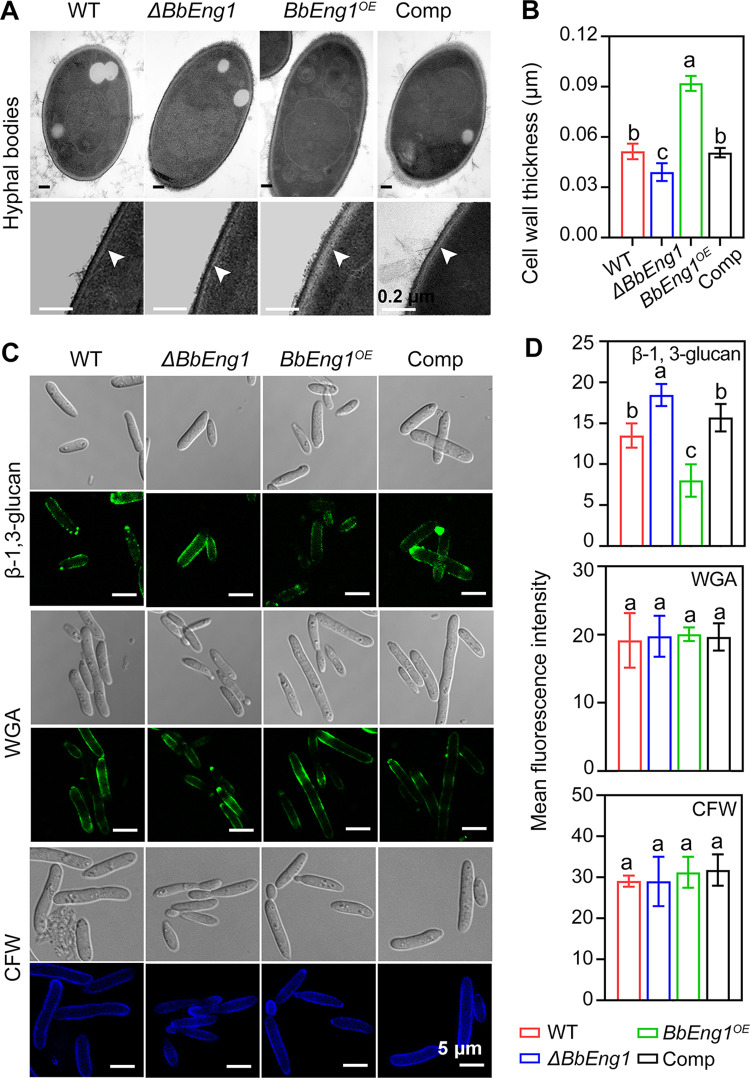
Cell wall structures of *B*. *bassiana* wild type and mutant strains. **A, B.** TEM micrographs (scale: 0.2 μm) for cell walls of hyphal bodies and cell wall thickness (n = 30). **C, D.** Confocal microscopic images (scale: 5 μm) for cell wall β-1,3-glucan and chitin and measurements of their contents. β-1,3-glucan was labeled with monoclonal β-1,3-glucan specific antibody and goat anti-mouse IgG-FITC. Chitin was labeled with WGA or stained with CFW as detailed in the Methods section. Mean fluorescence intensities were quantified densitometrically using the ImageJ software. Error bars: SDs. Different letters indicate significant differences (*P* < 0.01 in LSD test).

Cell wall morphology was examined using transmission electron microscopy (TEM) as detailed in the Methods section. These data indicated an electron-dense brush-like structure present on the surface (edge) of *BbEng1*^*OE*^ hyphal bodies but absent in control strains, resulting in an increase in cell wall thickness for *BbEng1*^*OE*^ (0.092 ± 0.005 μm) as compared to wild type (0.051 ± 0.005 μm, n = 30, *P* < 0.01) (Fig 4A and 4B). Increased cell wall thickness was also noted in TEM images of *BbEng1*^*OE*^ conidia and blastospores (S5 Fig). In contrast, the *ΔBbEng1* mutant showed a reduction in surface brush-like structures coupled to decreased cell wall thickness (to 0.039 ± 0.005 μm, n = 30, *P* < 0.01) of hyphal bodies (Fig 4A and 4B). Measurement of cell wall β-1,3-glucan contents from cells grown in 1/4 SDY (for 48 h) using an aniline blue assay revealed that overexpression of *BbEng1* resulted in a significant reduction in β-1,3-glucan level (38%). No obvious differences were seen with respect to β-1,3-glucan content between the *ΔBbEng1* and wild-type strains (S6 Fig). Similar results were obtained via detection of fluorescence intensities after probing with a monoclonal β-1,3-glucan specific antibody followed by application of a goat anti-mouse IgG-FITC secondary antibody in examinations of conidia and blastopores after fungal cells were fixed in 3% (v/v) formaldehyde as detailed in Methods section. Cell wall chitin contents assessed using HCl (6 N) hydrolysis (as detailed in Methods section) also indicated no obvious differences in chitin content for any of the strains tested. Similarly, no differences were seen in fluorescence labeling of the cell wall using FITC-conjugated wheat germ agglutinin lectin (WGA, specificity for N-acetylglucosamine and N-acetylneuraminic acid residues) or in cell wall labeling using calcofluor white (CFW, S6 Fig). Cell wall glucan detection using a monoclonal β-1,3-glucan specific antibody and a goat anti-mouse IgG-FITC antibody revealed that β-1,3-glucan levels were dramatically decreased in the cell walls of *BbEng1*^*OE*^ hyphal bodies (40.7%, *P* < 0.01), but increased in *ΔBbEng1* hyphal bodies (36. 7%, *P* < 0.01) as compared to wild type (n = 200). However, no obvious difference was observed in chitin content of hyphal bodies from any of the tested strains as seen after labeling with either WGA or CFW (Fig 4C and 4D).

**Fig 5:**
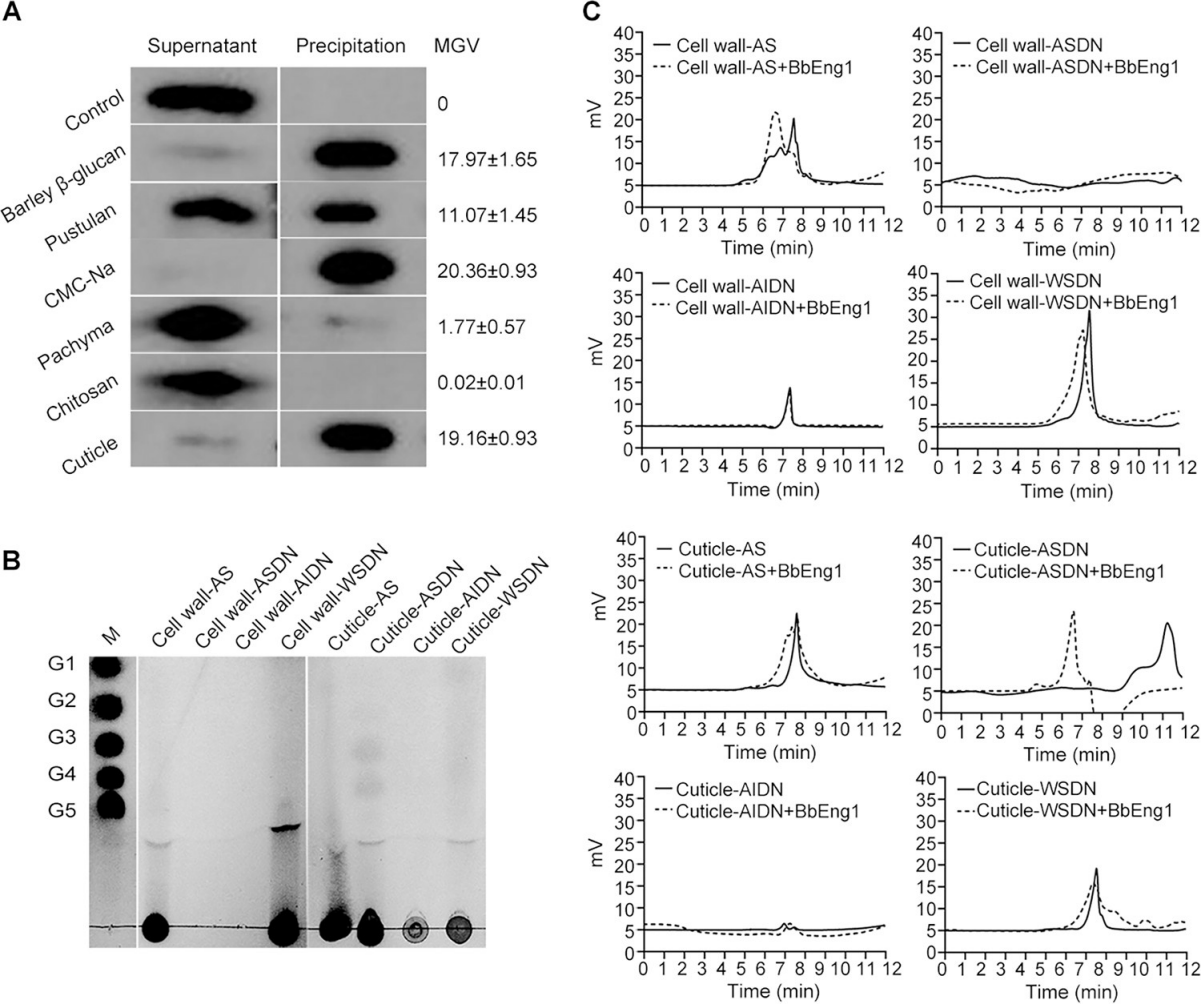
Binding of purified BbEng1 to various substrates. **A.** Western blots probed with anti-BbEng1 antibody after pull-down assays with indicated polysaccharides and silkworm cuticles as substrates (2%, w/v). The protein concentration of BbEng1 used in assays was 60 μg / mL. **B, C.** TLC and HPLC analyses for the carbohydrates released from the reaction of BbEng1 (2 μg, pH 6.0, 30°C for 1 h) with the extracts of polysaccharides (1%, w/v) from *B*. *bassiana* cell wall and silkworm cuticle. AS, alkali-soluble fraction. ASDN, WSDN and AIDN, alkali-soluble, water-soluble and alkali-insoluble fractions generated by nitrous deamination of fully de-*N*-acetylated alkali-insoluble fraction (AI).

Western blots probed with an anti-BbEng1 antibody and immunoprecipitation assays revealed binding of purified BbEng1 to barley β-glucan, carboxymethylcellulose sodium (CMC-Na), pustulan, and silkworm cuticles with lower binding to pachyman and little to no detectable binding chitosan (Fig 5A). Measurement of glucanase activity indicated release of reducing sugar from laminaritetraose (β-1,3-glucose), yeast glucan (β-1,3/1,6-glucose), barley β-glucan (β-1,3/1,4-glucose), and pachyman (β-1,3-glucose). No hydrolyzed products were detected when purified BbEng1 was tested against CM-cellulose (β-1,4-glucose), dextran (α-1,3/1,6-glucose), pustulan (β*-*1,6-glucose), chitosan (β-1,4-N-acetyl-D-glucosamine), or cellobiose (β-1,4-glucose) (S1 Table). BbEng1 displayed high glucanase enzyme activity towards yeast glucan at 30°C and pH 6.0, maintaining over 80% activity over a wide range of temperatures (20–70°C) and pH (3–6) (S7 Fig). Thin-layer chromatography (TLC) analyses indicated release of carbohydrate polysaccharides with different molecular weights, between mono- and penta-saccharides from yeast glucan, barley β-glucan, pachyman and laminaritriose after BbEng1 treatment. No hydrolyzed products were seen when cellobiose was used as the substrate for BbEng1 (S8 Fig). High-performance liquid chromatography (HPLC) analyses also revealed similar hydrolysis products after BbEng1 treatment of yeast glucan, barley glucan, pachyman and laminaritriose (elution peaks of 6.16, 7.42, 6.52 and 5.85 min, respectively), with no products detected after BbEng1 treatment of pustulan, CM-cellulose, dextran, cellulose, insect cuticle or colloidal chitin (S8 Fig). BbEng1 displayed activity (endo-β-1,3-glucanase) activity against the substrate periodate-oxidized laminarin, but none to the substrate 4-nitrophenyl-β-D-glucopyranoside (exo-β-1,3-glucanase activity) (S8 Fig).

Hydrolyzed products > pentasaccharides were seen by TLC analyses using purified fungal cell wall alkali-soluble fractions (AS) and water-soluble fraction (WSDN) as substrates, which were derived from nitrous deamination of fully de-*N*-acetylated alkali-insoluble fractions (AI) as detailed in the Methods section (Fig 5B). Similar hydrolyzed products were also seen after BbEng1 incubation with the AS and WSDN fractions as measured by HPLC (peaks eluted at 6.60 and 7.31 min, respectively) (Fig 5C). No products were seen after BbEng1 treatment of additional substrates including fully de-*N*-acetylated fungal AS (ASDN) or AI (AIDN) fractions (Fig 5B and 5C).

### BbEng1 alters cell wall carbohydrate epitopes on hyphal bodies

**Fig 6:**
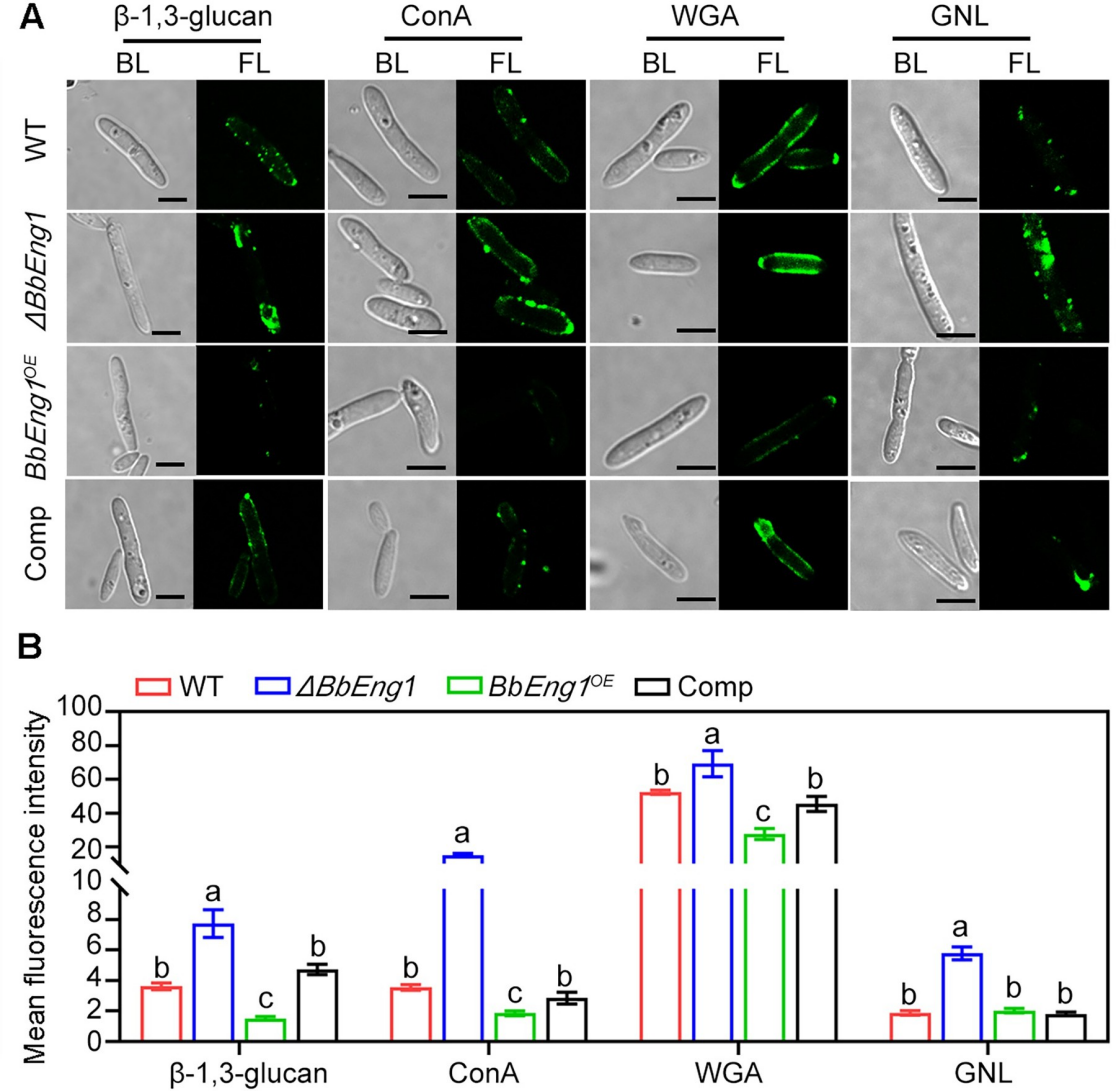
Carbohydrate epitopes and β-1,3-glucan levels on surfaces of *B*. *bassiana* wild type and mutant hyphal bodies. **A.** Fluorescence images (scale: 5 μm) of hyphal bodies treated with monoclonal β-1,3-glucan specific antibody and goat anti-mouse IgG-FITC and Fluor 488-labeled lectins. BL, bright light. FL, fluorescent light. **B.** Mean fluorescence levels quantified from at least 100 individual cells of tested strains. Error bars: SDs. Different letters indicate significant differences (*P* < 0.01 in LSD test).

Carbohydrate cell wall epitopes of *B*. *bassiana* hyphal bodies derived from the wild type, *ΔBbEng1*, and *BbEng1*^*OE*^ strains were further examined via use of three fluorescence-labeled lectins including: concanavalin A (ConA, specific to α-mannopyranosyl and α-glucopyranosyl residues), WGA and *Galanthus nivalis* lectin (GNL, specific to α-1,3-mannose), as well as by Western blots using a monoclonal β-1,3-glucan antibody as the probe. Significantly increased fluorescence signals were detected in *ΔBbEng1* hyphal bodies probed with ConA (3.3-fold, *P* < 0.01), WGA (0.3-fold, *P* < 0.01), GNL (2.1-fold, *P* < 0.01), and the β-1,3-glucan antibody (1.1-fold, *P* < 0.01) as compared to wild type, whereas *BbEng1*^*OE*^ hyphal bodies displayed decreased signals when probed with ConA, WGA, or the β-1,3-glucan antibody (0.4–0.5-fold, *P* < 0.01), but with little change in the GNL lectin-mediated signal (Fig 6). When tested against blastospores, both ConA and WGA labeling was more intense than GNL labeling, where the ConA and GNL fluorescence intensities on *BbEng1*^*OE*^ strain was significantly lower than other strains. For conidia both ConA and GNL labeling was more intense than WGA, however, the fluorescence intensities of all three lectins labeled *BbEng1*^*OE*^ strain were significantly lower than other strains. Although little to no labeling of conidia or blastospores was seen using the β-1,3-glucan monoclonal antibody for any of the strain, the fluorescence intensities on *BbEng1*^*OE*^ strain were significantly lower than those on the other strain (S9 Fig).

### BbEng1 expression facilitates fungal infection

**Fig 7:**
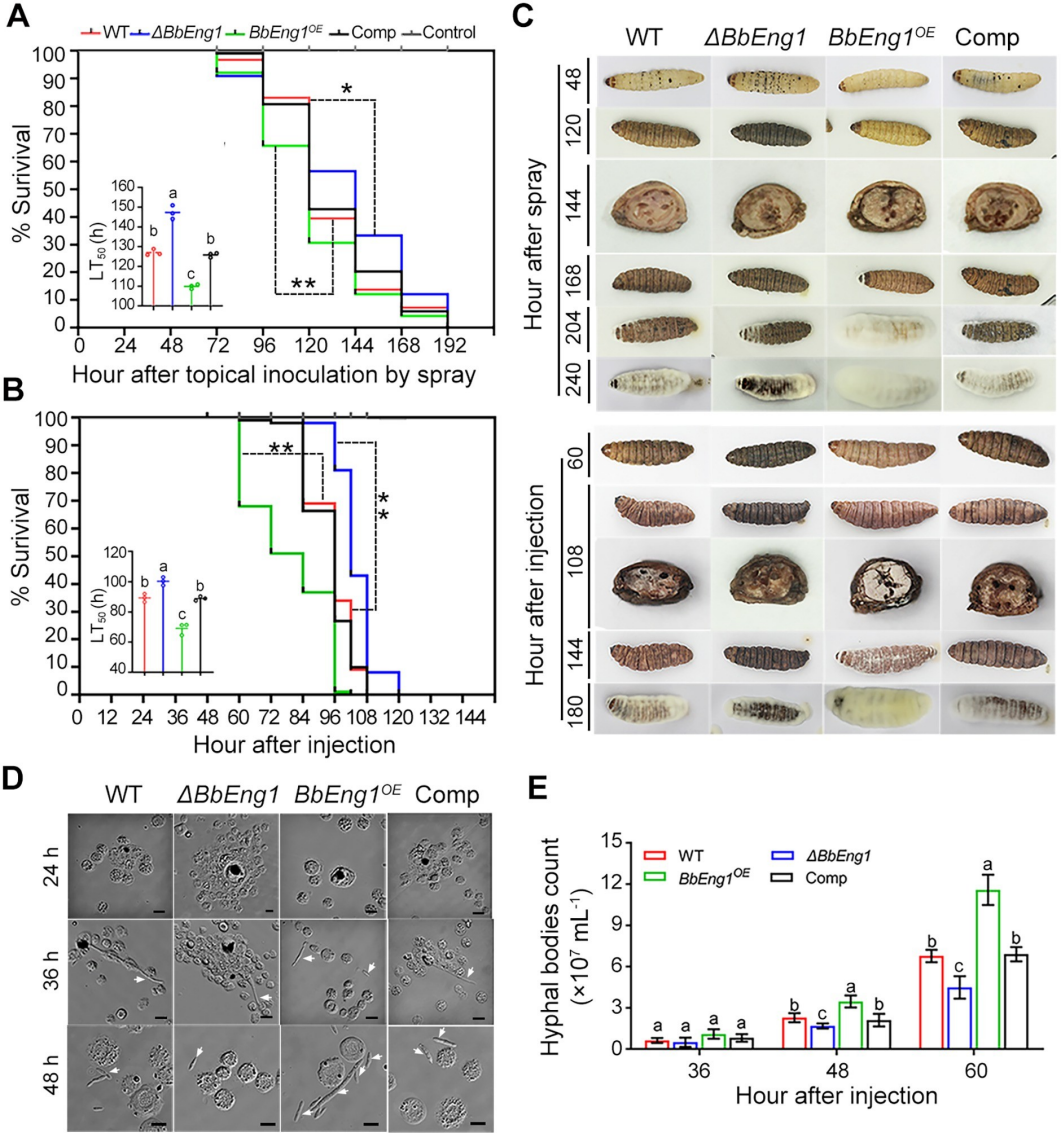
Virulence of *B*. *bassiana* wild type and mutant strains and insect immune responses. **A, B**. Survival trends and calculated LT_50_ values using *G*. *mellonella* larvae after topical application and intrahemocoel injection of conidial suspension. *P* < 0.05* or 0.01** in a log rank test. **C.** Symptoms of *G*. *mellonella* larvae infected by indicated strains and fungal outgrowth on cadavers at indicated time points. **D.** Microscopic images (scale: 5 μm) of fungal development and insect immune responses at indicated time points after injection. Arrows indicate fungal cells (hyphal bodies, HBs). **E.** Quantification of fungal biomass in hemolymph samples at 36, 48 and 60 h after injection as determined using qPCR analysis. Error bars: SDs. Different letters in **A**, **B** and **E** indicate significant differences (*P* < 0.01 in LSD test).

TLC and HPLC analyses revealed the release of detectible carbohydrate products from insect cuticle fractions (AS, ASDN and WSDN) after treatment with purified BbEng1 (Fig 5B and 5C), suggesting a potential role of the secreted BbEng1 in participation in degradation and utilization of host carbohydrate containing substrates during infection. Insect bioassays using last-instar larvae of the greater wax moth, *G. mellonella*, via topical application of the fungal strains revealed a calculated median lethal time to kill 50% of hosts (LT_50_) for the *BbEng1*^*OE*^ strain = 109.9 ± 1.2 h, which was significantly lower (*i*.*e*., showed increased virulence, *P* < 0.01) as compared to the wild type and complemented strains (LT_50_ = 127.0 ± 1.6 h and 125.8 ± 1.2 h, respectively) which were in turn significantly lower (*P* < 0.01) than the *ΔBbEng1* strain, LT_50_ = 147.3 ± 3.5 h (Fig 7A). Similarly, bioassays performed in which fungal cells were directly injected into host larvae (*i*.*e*., via cuticle-bypassing intrahemoceol injection) revealed a decreased LT_50_ for the *BbEng1*^*OE*^ strain (69.1 ± 3.9 h, *P* < 0.01) as compared to control wild type and complemented strains (89.3 ± 2.7 h and 88.9 ± 1.4 h), which were lower than the *ΔBbEng1* LT_50_ which was = 100.3 ± 2.7 h (*P* < 0.01) (Fig 7B). In addition, the *BbEng1*^*OE*^ strain proliferated more rapidly out of the cadavers as compared to the other strains (Fig 7C).

Melanization reactions/spots, hallmarks of one aspect of the insect immune system, were more apparent on *ΔBbEng1* infected larvae, 48 h after topical inoculation, and reduced on *BbEng1*^*OE*^ infected larvae, as compared to controls (Fig 7C). Larvae infected by *ΔBbEng1* turned black 120 h after topical inoculation and 60 h post-injection, retaining some of this color even during “mumification”, *i*.*e*., fungal out growth at 240 / 180 h for topical / injection applications as contrasted with the *BbEng1*^*OE*^ and control strains (Fig 7C). To examine host hemocyte-fungal interactions, conidia injected into the hemocoel were examined microscopically over a time-course of the infection. Unlike as seen in controls, fungal cells from *ΔBbEng1* encapsulated by hemocytes at 24 h post-injection did not escape encapsulation until 36–48 h post-injection, whereas *BbEng1*^*OE*^ conidia / fungal cells were not encapsulated by insect hemocytes even at the early stage post-injection, and were able to germinate and proliferate directly within the hemolymph without any delay (Fig 7D). Total fungal load analyses by qPCR from hemolymph samples of injected larvae revealed more rapid proliferation of *BbEng1*^*OE*^ than of wild type in the hemocoel, with the latter forming 50% and 71% more cells at 48 and 60 h post-injection, respectively. In contrast, proliferation of *ΔBbEng1* was reduced by 25–35% at the same time points in comparison to controls (Fig 7E).

**Fig 8:**
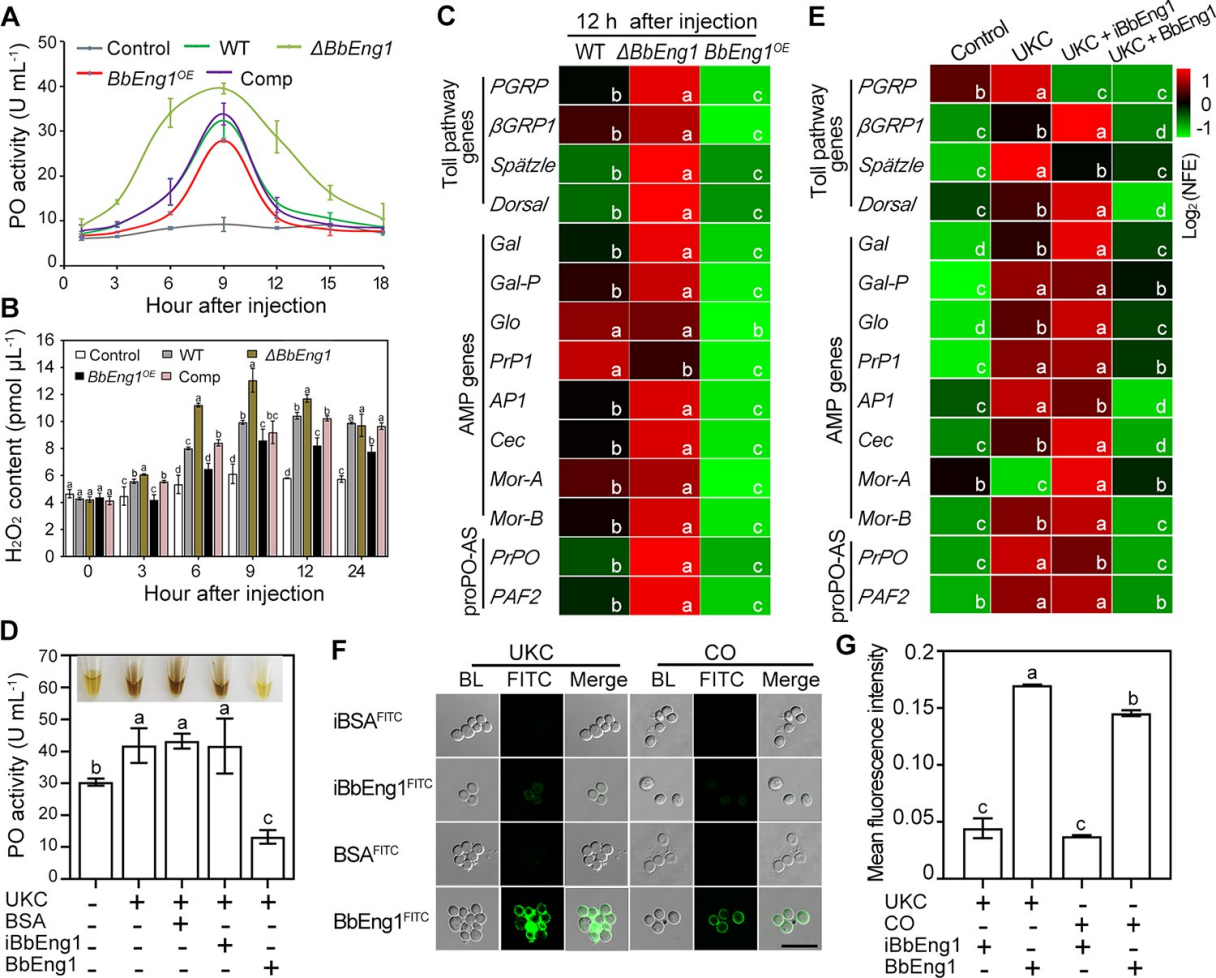
Immune responses of *G*. *mellonella* after challenge by *B*. *bassiana* wild type and UV-killed cells with or without addition of purified BbEng1. **A.** The phenoloxidase (PO) activities at indicated time points after injection. **B.** H_2_O_2_ (ROS) levels during fungal infection. **C.** Expression levels of genes involved in the Toll pathway, antimicrobial peptide (AMP) and proPO system in the larvae 12 h post-injection (*β-actin* used as a reference for normalized fold expression, NFE). **D.** Effects of added 0.5 μM BbEng1 or iBbEng1 (heated-inactivated BbEng1) on fungal cell wall (UKC, UV-killed *B*. *bassiana* conidia, 5 × 10^6^ conidia / mL)-activated PO activity (total volume: 2 μL). Hemolymph samples taken at 12 h after injection of UKC, UKC + BbEng1 or UKC + iBbEng1 were used for PO assay. Larvae injected with 2 μL of 0.85% NaCl or UKC+ BSA (bovine serum albumin, 0.5 μM) were used as controls. **E.** Expression levels of genes in the Toll pathway components, AMP proPO-system-associated genes in the larvae at 12 h after injection of UKC or UKC + proteins (*β-actin* used as the reference). **F, G.** Microscopic images and fluorescence intensities of UKC and conidia (CO) (n > 100) labeled with FITC-labeled BbEng1. Error bars: SDs. Different letters indicate significant differences (*P* < 0.01 in LSD test).

To examine host immune responses, phenoloxidase (PO) activity, reactive oxygen species (ROS) production, and expression levels of host Toll pathway genes were measured. For wild type, PO activity increased during the first 9 h period post-injection and then decreased (Fig 8A). In contrast, larval PO activity was significantly higher (*P* < 0.01) after injection of *ΔBbEng1*, and lower (*P* < 0.01) after injection of the *BbEng1*^*OE*^ strain (Fig 8A). ROS levels increased from 3 to 24 h post-challenge by the wild type, a response which was dampened for *BbEng1*^*OE*^ but higher for the *ΔBbEng1* strain (*P* < 0.01) (Fig 8B). Transcriptional profiling targeting 4 Toll pathway genes [25,26], 8 antimicrobial peptide (AMP) genes [16] and 2 proPO system-associated genes [27,28] were performed using larvae 12 h post-injection with the various strains as detailed in the Methods section. Expression levels for the Toll pathway genes (28–393%), 6 AMP genes (12–65%) and 2 proPO system-associated genes (56% and 68%) were higher for the *ΔBbEng1* versus wild type injected larvae (Fig 8C), whereas expression of all analyzed genes was lower in larvae injected with *BbEng1*^*OE*^ (14–99.8%, *P* < 0.01) (Fig 8C).

The effects of purified BbEng1 protein (2 μL of 0.5 μM BbEng1 per larvae) and / or ultraviolet (UV)-killed conidia (UKC, 2 μL of 5 × 10^6^ conidia / mL per larvae) on insect immune responses were examined. PO activity was induced by UKC injection, but dramatically repressed by inclusion of BbEng1 (55%, *P* < 0. 01). However, the UKC-activated PO activity was not repressed by injection with inactivated / denatured BbEng1 (iBbEng1) (Fig 8D). Transcriptional profiling revealed that the inclusion of BbEng1 also repressed UKC-activated expression of several host immune genes with the exception of *PGRP*, a Toll pathway gene whose expression was also decreased with the addition of iBbEng1 (Fig 8E). BbEng1 (FITC-labeled) was able to bind the cell walls of UV-killed as well as wild type (normal) conidia, whereas FITC-iBbEng1 only weakly bound to the conidial cell walls (Fig 8F and 8G).

### Glucanase homologs play similar roles in *Metarhizium* spp

Homologues of *BbEng1* were identified in the generalist *M*. *robertsii* (*MrEng1*, GCA_000187425.2) and the specialist *M*. *acridum* (*MaEng1*, GCA_000187405.1) fungal entomopathogens (S10 Fig). Similar to *BbEng1*, both *MrEng1* and *MaEng1* were mainly expressed in hyphal bodies proliferating in insect hemolymph, with *in vitro* expression requiring induction by insect-derived nutrients (cuticle or hemolymph extracts), as well as low levels of induction seen under osmotic and oxidative stress growth conditions (S10 Fig). To probe the effects of either homolog in mediating protective immune evasion, (1) *M*. *robertsii* strains overexpressing *BbEng1* (*Mr-BbEng1*^*OE*^) or *MrEng1* (*MrEng1*^*OE*^) and (2) *M*. *acridum* strains overexpressing *BbEng1* (*Ma-BbEng1*^*OE*^) and *MaEng1* (*MaEng1*^*OE*^) were constructed (S11 Fig). Similar to the *BbEng1*^*OE*^ strain, the various *Eng1* overexpression strains showed faster colony growth, more biomass accumulation, higher conidial yield, and accelerated germination as compared to their respective parental wild type strains (S12 Fig).

**Fig 9:**
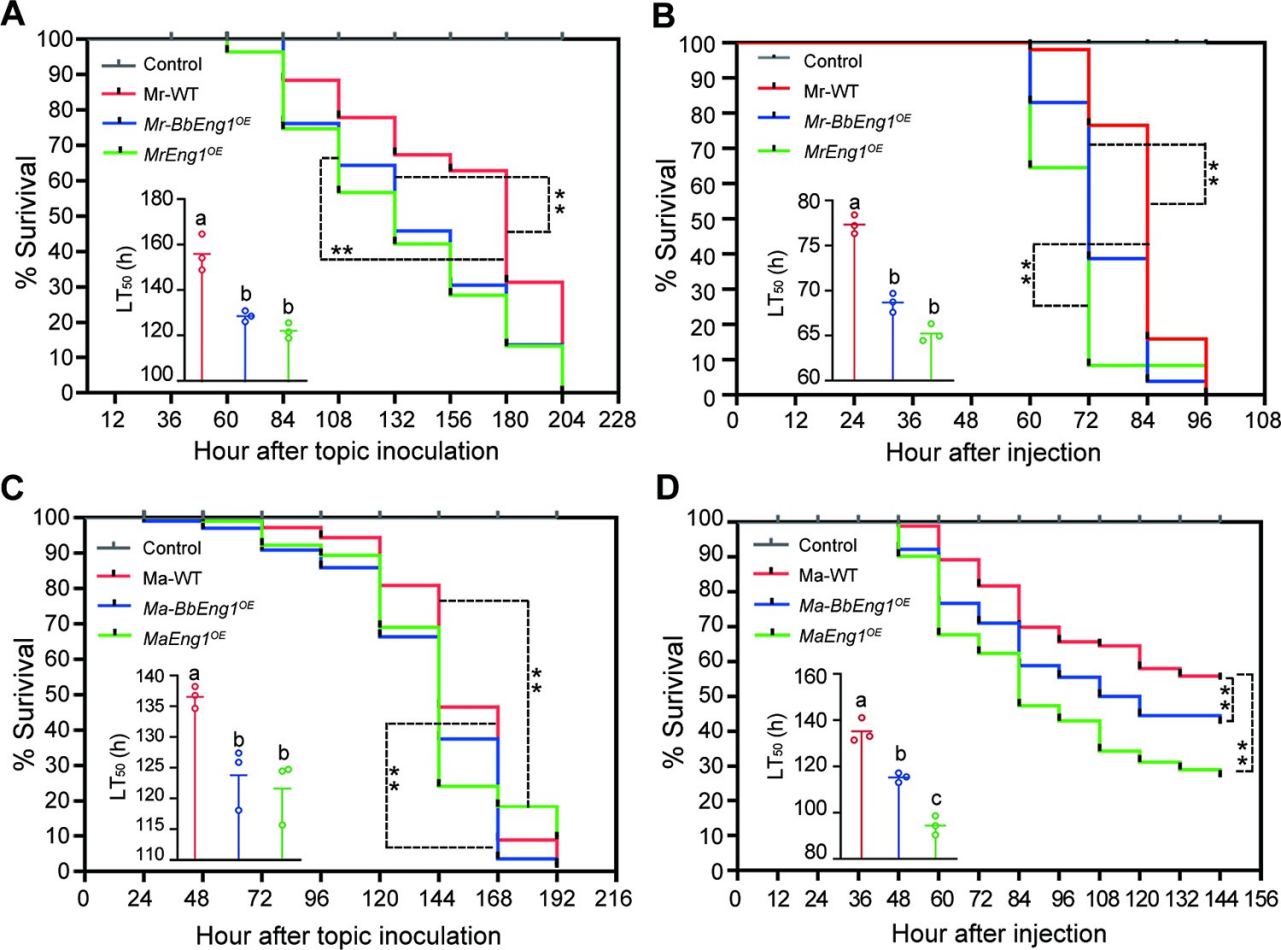
Insect bioassays of *M*. *robertsii* and *M*. *acridum* wild type and *Eng1* overexpression strains. **A, B and D.** Survival trends of *G*. *mellonella* larvae after topical application (A) or hemocoel injection (B and D). **C.** Survival trends of *L*. *migratoria manilensis* nymphs after topical inoculation. **P < 0.01 in log-rank test. *Mr-BbEng1*^*OE*^ and *MrEng1*^*OE*^, *M*. *robertsii* strains overexpressing *BbEng1* and *MrEng1*. *Ma-BbEng1*^*OE*^ and *MaEng1*^*OE*^, *M*. *acridum* strains overexpressing *BbEng1* and *MaEng1*. Marked LT_50_ estimates differ significantly (*P* < 0.01 in LSD test).

In addition, insect bioassays using *G*. *mellonella*, indicated significantly increased virulence for the *Mr-BbEng1*^*OE*^ and *MrEng1*^*OE*^ strains as compared to the wild type parent. The resultant LT_50_ values via topical application and intrahemocoel injection were 128.5 ± 2.3 and 68.7 ± 1.0 h for *Mr-BbEng1*^*OE*^, 122.0 ± 3.4 and 65.2 ± 1.0 h for *MrEng1*^*OE*^ and 155.9 ± 8.1 and 77.3 ± 1.0 h for wild type *M*. *robertsii* (Fig 9A and 9B). As *M*. *acridum* is a more acridid specific pathogen (although it can also infect *G*. *mellonella*), these strains were assayed using 5th instar locusts (*Locusta migratoria manilensis*) by placing 5 μL of a 10^7^ conidia / mL suspension on the host pronotum. LT_50_ values for *Ma-BbEng1*^*OE*^, *MaEng1*^*OE*^ and wild type *M*. *acridum* were 123.8 ± 5.0, 121.6 ± 5.2 and 136.6 ± 1.8 h, respectively (Fig 9C), indicating significantly increased virulence for the former strains as compared to the wild type parent (*P* < 0 .01). Insect bioassays performed *via* intrahemoceol injection of *G*. *mellonella* larvae gave similar results, with a calculated LT_50_ = 115.3 ± 2.0 h for *Ma-BbEng1*^*OE*^, 94.5 ± 4.2 for *MaEng1*^*OE*^ and 135.2 ± 5.1 h for wild type *M*. *acridum*, respectively (Fig 9D).

## Discussion

Glucanases produced by some plant and animal fungal pathogens during infection and/or in response to host signals, such as carbon sources, oxygen limitation or hypoxia, and iron, can remodel the cell wall to reduce or mask exposed PMAPs that comprise β-glucans, so as to evade host immune recognition [29–32]. The present study adds the existence of similar mechanisms in insect-pathogenic fungi including *B*. *bassiana*, and both generalist and more specialized *Metarhizium* species. Our data show these pathogens express a specific virulence-associated endo-β-1,3-glucanase (Eng1) during entry into the hemocoel as part of the infection process that can be mimicked *in vitro* by addition of insect-derived substrates including cuticle and hemolymph. In addition, a low level of induction can occur *in vitro* under osmotic or oxidative stress. Both of these occur during the infection process, both during initial stages of infection on the insect cuticle as well as during growth within the host hemocoel, and hence these may act minor cues for the fungus that they are interacting with a potential host [22,33]. Our hypothesis was that the main function of BbEng1 is to facilitate evasion of host immune activation during fungal infection by decreasing surface glucan that are recognized by host immune surveillance systems. Our data show that the protein is distributed on both cell wall and surface fractions. BbEng1 is secreted and can bind to the fungal cell wall. BbEng1 was previously found as a cell surface protein on hyphal bodies that could be released from cells by treatment with 2 mM DTT [21]. Here we show using a green fluorescence fusion protein BbEng1::GFP, distribution of the protein on the surface of hyphal bodies that was also labile to DTT treatment. These results suggest important disulfide linkages that may mediate BbEng1 interaction with other cell wall proteins and/or structures. Immunofluorescence (IFL) assays, however, showed punctate BbEng1 signals that distributed on the outside edge of/ in calcofluor white staining in hyphal bodies. This discrepancy is likely due to technical differences between the assays in which IFL experiments involve extensive washing as well as potential steric effects due to use of antibody/secondary antibody visualization.

To determine the role of BbEng1, genetic characterization including construction of a targeted gene knockout mutant (*ΔBbEng1*) and overexpression strains (*BbEng1*^*OE*^) were performed. Our data revealed that the *ΔBbEng1* strain was compromised in virulence whereas the *BbEng1*^*OE*^ showed enhanced virulence, using insect bioassays performed either via topical (cuticle) or intrahemocoel injection (cuticle-bypassing) methodologies. In larvae infected by *BbEng1*^*OE*^, cuticle melanization spots were reduced indicating a failure of the host to encapsulate the fungal cells by aggregated hemocytes. In contrast, infection by *ΔBbEng1* saw increased levels of cuticle melanization and prolonged encapsulation of the fungal cells by aggregated hemocytes, which was consistent with these cells having a reduced ability to escape these responses. Overall immune responses followed a similar pattern, with host PO activity and expression of suite of immune genes higher after *ΔBbEng1* infection but lower after *BbEng1*^*OE*^ infection as compared to the wild type. Insect glucan-binding proteins (β-1,3-glucan recognition proteins, βGRPs) can recognize fungal PAMPs, *i*.*e*., β-1,3-glucan and chitin [34,35] to activate innate immune response including activation of the prophenoloxidase (proPO) cascade [36,37] and the Toll receptor pathway to initiate production of antimicrobial compounds and other immune responses [38]. As mentioned, expression of the host *β-GRP* gene as well as other Toll pathway genes and proPO cascade genes was lower during *BbEng1*^*OE*^ infection but higher during *ΔBbEng1* infection as compared to wild type, correlating with the altered insect immune responses observed in the bioassays. All of these responses are hypothesized to be mainly due to decreased levels of immune recognized cell wall glucan in the *BbEng1*^*OE*^ as compared to increased levels seen for the *ΔBbEng1* strain, when compared to the wild type parent. However, additional altered cell wall epitopes as detected using lectins (ConA and WGA) and anti-β-1,3-glucan antibody were noted. These data revealed exposure of other potential PAMPs in hyphal bodies, including α-mannopyranosyl / α-glucopyranosyl residues, GlcNAc and GlcNAc acid residues (chitin) and β-1,3-glucan were significantly increased in the *ΔBbEng1* strain, and conversely decreased in the *BbEng1*^*OE*^ strain as compared to wild type. Thus, in addition to affecting β-1,3-glucan levels, modification of other surface PAMPs may occur in the mutant strains due to changes in surface glucan levels. Purified BbEng1 was shown to be able to bind to the cell walls of both UV-killed (UKC, with our data showing that these cells can still activate host immune responses) and normal conidia, with the UKC-activation of host immune responses blocked by co-injection with purified BbEng1 but not by denatured BbEng1. These data support the idea that BbEng1 removes surface glucans rendering the cells more “stealth-like” in terms of being recognized by host immune surveillance systems. These findings are similar to previous observations in mammalian fungal pathogens *H*. *capsulatum* and *C*. *albicans*, which secrete endo-β*-*1,3-glucanase (Eng1) and an exo-β-1,3-glucanase (Xog1), respectively, during infection or in response to various host-derived signals that act to remove cell wall β-glucans facilitating evasion of the fungal cells from host immune recognition [29–32].

Aside from evasion, assimilation of host-derived nutrients is crucial for successful fungal infection. Phytopathogenic fungi are known to secrete β-1,3-glucanases and other plant cell wall hydrolases and glycosidases, including polygalacturonases, cellulases, xylanases, laccases, and hemicellulases, to break down plant cell wall structures for acquisition of host nutrients and facilitation of fungal colonization on plant tissues during infection [39,40]. Similarly, fungal insect pathogens express a variety of hydrolytic enzymes, such as proteases, chitinases, lipases and peptidases, to breach the insect exoskeleton for uptake of host nutrients [4,41,42]. Different, but overlapping, sets of enzymes are presumably important for breaching the cuticle and the fungal growth in the hemocoel. It is unclear the extent to which BbEng1 may act in terms of nutrient acquisition and its main function is likely to be to act on the surface of the fungal cell. Indeed, cell wall visualization of hyphal bodies revealed that *BbEng1*^*OE*^ cells show increased cell wall thickness (and decreased β-1,3-glucan) whereas the *ΔBbEng1* mutant displayed the opposite phenotype (decreased cell wall thickness and increased β-1,3-glucan levels) when compared to the wild type. It is well known that β-1,3-linked glucan is a main component of fungal cell walls, and β-1,3-glucanases function in cell wall glucan metabolism, impacting fungal growth and development, including morphogenesis during spore germination, hyphal elongation and hyphal branching in filamentous fungi [43–47]. Thus, it is not too surprising that the *BbEng1*^*OE*^ strain showed faster growth, abnormal hyphal morphologies, increased conidiation levels, faster germination, mono-directional hyphal growth as compared to the wild type. As expected, *BbEng1*^*OE*^ conidia and blastospores had thicker cell walls and lower β-1,3-glucan content. However, cell wall composition was not significantly altered for *in vitro* cells (as opposed to the *in vivo* hyphal bodies) when comparing *ΔBbEng1* to the wild-type strain. No activity for BbEng1 was seen using insect cuticle or chitin (colloidal chitin) as substrates, however, release of carbohydrate products from insect cuticle fractions (AS, ASDN and WSDN) after treatment with BbEng1 were detected by TLC and HPLC analysis. These data suggest a potential role for secreted BbEng1 in degrading insect β-glucan substrates, however the extent to which this occurs and its importance in the infection process remains to be further characterized.

Our data shows that BbEng1 helps fungal cells to evade host immune responses, including hemocyte encapsulation and melanization likely by depleting the fungal cell surface of a crucial PAMPs (β-1,3-glucan) that is a critical step in the transition from hyphal invasion into the hemocoel to the formation of free floating yeast-like hyphal bodies that must evade host detection and/or any subsequent immune responses. Our data show that this process occurs in *Metarhizium* and likely in most if not all fungal entomopathogens. Overall, our results determine that the endo-β-1,3-glucanase BbEng1 secreted during the infection process of *B*. *bassiana* and other fungal entomopathogens acts to render fungal cells less able to be recognized by host immune systems. BbEng1 functions as a cell wall remodeler enabling removing cell wall glucans, allowing for fungal growth in host hemocoel.

## Materials and methods

### Fungal and bacterial strains and insects

Wild type *Beauveria bassiana* strain Bb2860 (CGMCC 7.34), *Metarhizium acridum* CQMa 102 and *M*. *robertsii* ARSEF23, and their derived genetically modified strains were routinely cultured on potato-dextrose agar/broth (PDA/PDB) and Czapek-Dox agar / broth (CZA / CZB). For examination of growth and conidiation, fungal strains were cultured on 1/4 SDAY (1:4-diluted Sabouraud dextrose agar supplemented with 1% (w/v) yeast extract), PDA, CZA, and CZA in which the sucrose carbon source was replaced with glucan, trehalose, glucose, mannose, erythritol, galactose or fructose at 3% (w/v). Plates were inoculated by placing 2 μL of conidial suspension (10^7^ spores / mL) on the center of plates and incubating the plates for 3–14 days at 26°C with a photoperiod of 15 L: 9 D. The yeast *Pichia pastoris* strain GS115 (Invitrogen, Carlsbad, USA) was used for heterologous expression of BbEng1. *Escherichia coli* DH5α and *Agrobacterium tumefaciens* AGL-1 were employed for DNA manipulations and fungal transformations, respectively.

Last instar larvae of *G*. *mellonella* (greater wax moth) from Huiyude Technology (Tianjin, China) and the 5th instar nymphs of *L*. *migratoria manilensis* provided by School of Life Sciences, Chongqing University (China), were used in virulence bioassays and insect immune responses.

### Targeted gene manipulation

All primers used for gene manipulation are given in S2 Table. To examine BbEng1 gene expression, the enhanced green fluorescent protein (eGFP) gene was fused to the promoter sequence of *BbEng1*. Briefly, the *BbEng1* promotor (1093 bp) was amplified from *B*. *bassiana* using primer pair C1 / C2 and inserted into the *Hin*dIII / *Eco*RV restriction sites of pK_2_surPB3-GFP [21] to form vector PBbEng1::GFP. The resultant vector was introduced into wild type *B*. *bassiana* using *Agrobacterium*-mediated transformation of conidia and selection of 40 μg / mL sulfonylurea [48]. Transformants were verified using PCR with primer C1 / GFP2 (1823 bp). For protein localization, a strain expressing the BbEng1::GFP fusion protein was constructed by fusing *eGFP* to 3’-terminus of *BbEng1*. Briefly, *BbEng1* (4168 bp containing 2194 bp promotor sequence) was cloned into the *Nde*I / *Eco*RV restriction sites of pBARGPE1-GFP [21] replacing *gpdA* promoter to yield vector construct BbEng1::GFP, which was then transformed into wild type *B*. *bassiana* as described with selection of 200 μg / mL phosphinothricin [49]. Transformants were verified via PCR with primer pair RT1 / GFP2 (1160 bp).

For construction of a targeted gene knockout of *BbEng1*, its partial coding region (197 bp) was replaced with the *bar* marker (resistant to phosphinothricin) through *A*. *tumefaciens*-mediated homologous recombination as described previously [50]. Putative gene disruption mutants were screened on selective media (200 μg / mL phosphinothricin) and examined via PCR with primer pair S1 / S2. Integrity of mutants were then verified using reverse transcription-PCR (RT-PCR) with primer pair RT1 / RT2 (to check transcript levels) and Southern blotting (single insertion in correct site). Southern blots were performed using a digoxin-labeled *bar* fragment as probe. Complementation strains of *BbEng1* were generated by ectopic integration of *BbEng1* into the *ΔBbEng1* mutant. Briefly, the coding region and upstream promoter regions of *BbEng1* (4118 bp) was amplified from the wild-type DNA using primer pair RC1 and RC2 and inserted into the *Bam*HI / *Xba*I restriction sites of modified pCB1536 containing the *sur* (resistant to sulfonylurea) marker [51]. The resultant vector was introduced into the *ΔBbEng1* strain as described above. Transformants were selected on plates amended with 40 μg / mL sulfonylurea, screened by PCR with primer pair S1 / S2, and verified by RT-PCR with primers RT1 / RT2. Southern blotting was performed to confirm the integration of *BbEng1*. A *BbEng1* overexpression strain was constructed by an introduction of its coding sequence under control of the *Aspergillus nidulans gpd* promoter. Briefly, the *BbEng1* coding region (1947 bp) was amplified with primer pair OE1 / OE2 from the wild-type DNA and cloned into the *Bam*HI / *Eco*RI restriction sites of pBARGPE1. The resultant vector was then transformed into the wild type strain. Transformants were selected on plates containing 200 μg / mL phosphinothricin, and verified by PCR using primer pair S3 / S4 (partial *PgpdA*::*BbEng1* fragment, 652 bp) and RT-qPCR with the primer pair RT1 / RT2.

To overexpress *BbEng1* and its homologous gene, *MrEng1* (GCA_000187405.1) in *M*. *robertsii*, and *MaEng1* (GCA_000187425.2) in *M*. *acridum*, the coding sequences of *BbEng1*, *MrEng1* and *MaEng1* were amplified from *B*. *bassiana*, *M*. *robertsii and M*. *acridum* using primer pairs OE3 / OE4, OE5 / OE6 and OE7 / OE8, respectively, and inserted into *Bam*HI and *Eco*RV restriction sites of pK2surPB3-GFP [12] replacing the *GFP* sequence and leading to expression of each target gene controlled by the *B*. *bassiana* constitutive promoter PB3 (glyceraldehyde-3-phosphate dehydrogenase gene (*Bbgpd*) promoter). The resultant vectors were introduced into *M*. *robertsii* and *M*. *acridum* via *A*. *tumefaciens*-mediated transformation [48], followed by selection of 200 μg / mL phosphinothricin. Transcript levels of *BbEng1*, *MrEng1* or *MaEng1* after growth in PDB (48 h) and analyzed by RT-qPCR with primer pairs RT1 / RT2, RT3 / RT4 and RT5 / RT6. The endogenous gene *gpd* of each fungus was used a reference.

### Gene expression and blotting analyses

RT-PCR and RT-qPCR analyses and/or analysis of fluorescence signals in the PBbEng1::GFP strain were performed to examine the expression patterns of *BbEng1* and its’ its homologous genes, and insect immune genes. RT-PCR and RT-qPCR analyses were performed toward target genes and the reference of *18S rRNA* in *B*. *bassiana* (XM_008603008.1), *gpd* in *M*. *robertsii* (XM_007825673.1) and *M*. *acridum* (XM_007817733.1), and *β-actin* (XM_026904349) in *G*. *mellonella*. Primers for detection of all genes examined are given in S2 Table. Transcript levels of *BbEng1* in the *G*. *mellonella* hemolymph-derived fungal cells and hyphal bodies of the disruption, complementation, and overexpression strains were examined via RT-PCR and the used cDNA samples were prepared as described previously [52]. RT-qPCR and / or GFP fluorescence signals were used for transcriptional profiling of *BbEng1* and its homologues in the wild type and overexpression strains of *B*. *bassiana*, *M*. *robertsii* and *M*. *acridum*. Expression levels of host (*G*. *mellonella*) immune genes were also examined via RT-qPCR with primers (S2 Table). Conidia, blastospores, hyphal bodies, aerial hyphae and submerged hyphae were collected as described previously [21,52]. Media used included basic salt broth (BS, 0.3 g / L MgSO_4_.7H_2_O, 0.3 g / L K_2_HPO_4_, 0.3 g /L NaCl) [12], 1/4 SDY, BS containing insect-derived nutrients (*i*.*e*., 5 mL / L silkworm hemolymph or 0.167 g / L silkworm cuticle), and CZB amended with the carbon source of 1% chitin, glucan or trehalose. For response to stress cues, fungal growth was initiated on CZB supplemented with 0.5 M NaCl, 1.0 M sorbitol, 5.76 mM H_2_O_2_, 37 μM menadione, 0.78 mM tert-Butyl hydroperoxide or lactic acid (2%). For fungal growth under low oxygen conditions, fungal cells were inoculated on CZB broth sealed with liquid paraffin for static cultivation (with ~6% of initial O_2_ concentration) [53]. Insect hemolymph and cuticle were isolated and prepared from the 5th instar larvae of *Bombyx mori* as described previously [52]. To assess transcript levels of *BbEng1* and its homologues in the overexpression strains, RNA was isolated from 1/4 SDY (48 h) cultures. Insect immune genes (S2 Table) were examined at 12 h post-injection with 2 μL suspension aliquots of (i) conidia (10^7^ conidia / mL), (ii) UV-killed conidia (*i*.*e*., inactive conidia, UKC, 5 × 10^6^ spores / mL, killed by ultraviolet with an ultraviolet radiometer (BLX-254) at 254 nm with 8 J / cm^2^ [54], (iii) purified BbEng1 protein (0.5 μM) + UKC, and (iv) heat-inactivated BbEng1 protein (iBbEng1, 0.5 μM, treated at 100°C, 10 min) + UKC. Larvae injected with 2 μL of 0.85% NaCl were used as controls.

For Western blot analyses, two predicted BbEng1 antigenic regions, (RGTIPDRPDDNWSWV, residues 43–56 of the N-terminal domain of GH16 family glucanases; TAAPEPESANQIC, residues 574–586 of the C-terminal domain) were synthesized and conjugated with keyhole limpet hemocyanin. Targeted antibodies were raised in New Zealand White rabbits and designated as anti-BbEng1 antibody. Western blot analyses were performed according to the manufacturer’s instructions (Bio-Rad). Samples tested included BbEng1 secreted by the wild-type and overexpression strains grown in either insect-derived nutrient cuticle or hemolymph containing minimal media for 6 h and (iii) insect hemolymph for 48 h after injection of 2 μL 10^7^ spores / mL. Blots were probed with the anti-BbEng1 antibody as described previously [52]. Protein concentrations were quantified using a BCA (bicinchoninic acid) assay kit (Generay Biotech, Shanghai, China). BbEng1 concentrations were densitometrically quantified using the yeast expressed, purified BbEng1 as a standard and the ImageJ software [55]. Secreted BbEng1::GFP fusion protein was detected using similar protocols and blots probed with an anti-GFP antibody (Thermo-Fischer Science).

Southern blot analysis was performed using standard protocols with a DIG-High Prime DNA labeling and detection starter kit I (Roche). Probes corresponding to the *bar* and *sur* fragments were prepared as described previously [56] and used for verification of *ΔBbEng1* mutant (*bar*) and complementation strain (*sur*), respectively.

### Assays for phenoloxidase (PO) and reactive oxygen species (ROS)

PO activity in insects was assayed as described previously [57]. Samples included a time course of infection, *i*.*e*., 3, 6, 9, 12, and 18 h after intra-hemocoel injection of 2 μL conidial suspension (10^7^ conidia / mL), as well as at 12 h after intra-hemocoel of (i) UV-killed wild-type conidia (UKC, 5 × 10^6^ spores / mL), (ii) purified BbEng1 protein (0.5 μM) + UKC, (iii) heat-inactivated BbEng1 protein (iBbEng1, 0.5 μM) + UKC. Larvae injected with 2 μL of 0.85%NaCl and UKC + 0.5 μM BSA were used as controls. ROS levels in insect hemolymph were examined by quantification of H_2_O_2_ content at 3, 6, 9, 12 and 24 h after intra-hemocoel injection of 2 μL conidial suspension (10^7^ conidia / mL) using the OxiRed Probe (BioVision) and a hydrogen peroxide colorimetric / fluorometric assay kit (BioVision) as described previously [12,58].

### Heterologous expression and characterization of BbEng1

To determine BbEng1 activity, the protein was expressed in *P*. *pastoris* and purified from the yeast culture. Briefly, *BbEng1* cDNA lacking the endogenous signal peptide was amplified by PCR with primer pair P1 / P2 and cloned into the *Eco*RI / *Not*I restriction sites of plasmid pPIC9K (Invitrogen), and the integrity of the clone was verified by sequencing. The resultant vector was introduced into the methylotrophic yeast strain *P*. *pastori*s GS115 (Invitrogen) according to the manufacturer’s instructions. Selection of the recombinants, protein expression and purification were performed as described previously [52]. Protein was quantified using the BCA assay.

The ability of BbEng1 to bind various polysaccharides, insect cuticle and the surfaces of fungal conidia was examined in pull-down assays followed by Western blot analyses as well as by using fluorescein isothiocyanate (FITC)-labeled proteins as described previously [15,59]. Bound protein detected in either assay was densitometrically quantified using the ImageJ software [55].

Glucanase activity was assayed using a variety of substrates including barley β-glucan, yeast glucan, pachyman, carboxymethyl cellulose (CM-cellulose), dextran, pustulan, colloidal chitin, laminaritetraose and cellobiose. For the slightly soluble or insoluble barley β-glucan, pachyman, CM-cellulose and pustulan, 10 mg aliquots of each substrate were soaked in 100 μL ethanol (100%) and mixed with 800 μL dd-H_2_O. The mixtures were incubated in a water bath at 100°C until the substrates were dissolved (barley β-glucan and pustulan) or formed sol like substance (CM-cellulose and pachyman) in 1 mL volumes of dd-H_2_O after cooling. The dinitrosalicylic acid (DNS) method [60] with slight modification [61] was used to quantify enzymatic activity. Briefly, 2 μg purified BbEng1 in 10 μL 0.02 M phosphate buffer saline (PBS, pH 7.2) was mixed with 90 μL mixture containing 10 mg / mL glucan substrate in 50 mM sodium acetate-acid acetate (NaAc-Ac) buffer (pH 6.0), and incubated at 37°C for 1 h. The mixture was heated at 100°C for 5 min after 200 μL DNS buffer (182.0 g / L C_4_H_4_KNaO_6_, 6.3 g / L C_7_H_4_N_2_O_7_, 21.0 g / L NaOH, 5.1 g / L C_6_H_5_OH, 5.1 g / L Na_2_SO_4_) [60] was added to terminate the reaction, and then cooled down on an ice bath for 3 min. The absorbance at 540 nm (OD_540_) of the supernatant was photometrically determined with glucose (reducing sugar) used as the standard as described [62]. Alternatively, a glucose oxidase method was used for assay of BbEng1 enzyme activity to laminaritetraose and cellobiose by measurement of produced glucose levels according to the manufacturer’s guides (Applygen Technologies Inc, China). One unit of β-glucanase activity was defined as the amount of enzyme required for a release of 1 μmol reducing sugar (glucose) per minute per μg of protein under the tested conditions. To reveal optimal temperature for BbEng1 activity, reaction mixtures of BbEng1 (2 μg) and yeast glucan (10 mg / mL) were incubated over a range of 20°C to 90°C at 10°C intervals. To determine enzymatic thermostability, 2 μg BbEng1 in 10 μL 0.02 M PBS (pH 7.2) was incubated for 1 h over a range of temperatures, followed by assessing enzymatic activity using yeast glucan at 30°C. The optimal pH of BbEng1 activity was determined at 30°C using yeast glucan as substrate in different buffers at pH 3.0 to 6.0 adjusted with 50 mM NaAc-Ac buffer and 7.0 to 9.0 adjusted with 50 mM Tris-HCl buffer. Relative enzyme activity was evaluated with respect to the maximal enzyme activity (100%) in a tested pH range. All the experiments were repeated three times with different batches of purified BbEng1 samples.

TLC and HPLC analyses were performed to quantify the products of substrates hydrolyzed by BbEng1. For TLC, 10 μL 0.02 M PBS (pH 7.2), 2 μg BbEng1 and 90 μL NaAc-Ac buffer (pH 6.0) containing 1% (w/v) of each substrate, namely yeast glucan, barley β-glucan, pachyman, CM-cellulose, pustulan, dextran, colloidal chitin, laminaritetraose or cellulose, were mixed and reacted at 30°C for 1 h, and the reaction was terminated by heating at 100°C for 5 min. Aliquots (5 μL) of reaction supernatants were examined on high temperature (110°C)-activated TLC Silica gel 60 F_254_ plates (Merck, Germany). The solvent used was n-butanol: methanol: water 8: 4: 3 (v/v/v). The plate was air-dried after resolution, and developed by spraying with 5% concentrated sulphuric acid (v/v), 0.5% vanillin (w/v) in ethanol, followed by heating at 95°C for 10-min color development [63]. Glucose, maltose, maltotriose, maltotetraose and maltopentaose were used as standards on the TLC plates. For HPLC, reaction supernatants were first filtered through a 0.22 μm filter. Aliquots (20 μL) of samples were analyzed using a sugar-H column (4.6 mm × 250 mm, 5 m) (Welch Co. Ltd., Shanghai, China) that was maintained at 80°C and coupled to an RI-201H detector. The mobile phase was composed of 5 mM H_2_SO_4_ at a flow rate of 0.6 mL / min.

For determining endo- vs exo-glucanase activities, periodate-oxidized laminarin and 4-nitrophenyl-β-D-glucopyranoside (p-NPG, Sigma) were used as substrates, respectively. Periodate-oxidized laminarin substrate preparation was performed as described previously [64] and the assay was carried out using the DNS method as described above. The substrate p-NPG was used to test for exo-1,3-β-glucanase using exo-1,3-β-D-glucanase (Megazyme) as a control, as described previously [65]. One unit of enzyme activity was defined as the amount of enzyme required for a release of 1 μmol p-nitrophenol per hour under standard assay conditions.

### Assays for β-1,3-glucan and chitin contents in fungal cell wall

An aniline blue assay was carried out to assess the content of cell wall β-1,3-glucan from fungal cells after growth in 1/4 SDY (48 h). Sample preparation and staining with aniline blue solution (0.067% aniline blue, 0.35 M HCl, 0.98 M glycine-NaOH, pH 9.5) were performed as described [66] via measurement of fluorescence at Ex / Em = 405 / 560 nm. The β-1,3-glucan analog curdlan (Sigma), was used as a β-1,3-glucan standard to calculate β-glucan content (μg / mg dry biomass). Levels of β-1,3-glucan in cell walls were also probed using a monoclonal β-1,3-glucan specific antibody (Biosupplies) and goat anti-mouse IgG-FITC (Proteintech). Briefly, fungal cells (hyphal bodies, conidia and blastospores) were fixed with 3% (v/v) formaldehyde at 65°C for 30 min after washing three times in 0.1 M PBS (pH 7.4). The fixed cells were washed and incubated with the monoclonal β-1,3-glucan specific antibody and goat anti-mouse IgG-FITC as described [67, 68]. The fluorescence of the stained cells was visualized with a confocal microscopy.

Cell wall chitin was determined as described [69]. Briefly, SDS-extracted cell walls (~ 5 mg dry weigh) hydrolyzed with 6 N HCl at 100°C for 17 h were evaporated at 65°C and resuspended in water (1 mL). After 0.1 mL 1.5 N Na_2_CO_3_ in 4% acetylacetone was added, the samples were incubated at 100°C for 20 min and then mixed with 0.7 mL of 96% ethanol. The mixture was reacted 1 h with 0.1 mL of solution containing 1.6 g of p-dimethylaminobenzaldehyde in 30 mL of concentrated HCl and 30 mL of ethanol, followed by reading the absorbance at 520 nm. Glucosamine was used as a standard for estimation of chitin content. Cell wall chitin was also assayed by fluorescent labeled wheat germ agglutinin (WGA) labeled using cells fixed with 3% (v/v) formaldehyde, and by calcofluor (CFW) staining as described [68,70].

### Extraction of glucan from fungal cell wall and carbohydrates from insect cuticle

Fungal cell walls and insect-derived polysaccharides were extracted as described previously [71], yielding: (i) alkali-soluble fraction (AS, including α-glucans and β-1,3/β-1,6-glucan); (ii) alkali-insoluble fraction (AI); (iii) water-soluble fraction generated by nitrous deamination of fully de-*N*-acetylated AI (WSDN, composed of β-1,3-glucan and β-1,6-glucan); (iv) alkali-soluble fraction resulting from nitrous deamination of fully de-*N*-acetylated AI (ASDN, β-1,3-glucan and β-1,6-glucan); and (v) alkali-insoluble fraction produced via nitrous deamination of fully de-*N*-acetylated AI (AIDN). Fungal cells were grown by shaking in 1/4 SDB for 2 days at 26°C. Fifth instar larvae cuticle of *B*. *mori* was isolated as described [52] and used for extraction of insect-derived polysaccharides.

### Microscopy

For confocal microscopy, an inverted confocal laser scanning microscope (SP8, Leica) with an argon ion laser was used. GFP and FM4-64-labeled membrane signals were visualized at the excision / emission wavelengths of 488 / 507 and 515 / 640 nm, respectively. Lectin binding assays to fungal cells were performed with Alexa Fluor 488-labeled lectins as described previously [8] and the manufacturer’s guides. The following lectins were tested: ConA, WGA (Molecular Probes-Invitrogen) and GNL (Vector Laboratories). β-1,3-glucan on fungal cell surfaces was detected with a monoclonal β-1,3-glucan specific antibody (Biosupplies, Parkville, Australia) and goat anti-mouse IgG-FITC (Proteintech) as described previously [12] and visualized at 488 / 530 nm. The cell wall components chitin and β-glucan were stained with CFW (Fluka, USA) and WGA, or probed with monoclonal β-1,3-glucan specific antibody and goat anti-mouse IgG-FITC, as described previously [67,68,70]. CFW fluorescence signals were shown at 405 / 435 nm.

IFL assays were performed as described previously [72]. Briefly, 1 mL aliquots of hemolymph-derived fungal cells (hyphal bodies, 2 × 10^6^ cells / mL) were fixed with 4% (v/v) paraformaldehyde for 30 min. After washing three times in precooled 0.1 M PBS (pH 7.4), samples were incubated with the blocking buffer (1% (w/v) bovine serum albumin (BSA) and 0.05% (v/v) Tween-20 in 0.01 M PBS, pH 7.4) at 22°C for 30 min. Fungal cells collected by centrifugation were labeled 1 h at 22°C with diluted anti-BbEng1 polyclonal antibody with the solution consisting of 5% (v/v) BSA and 0.1 (v/v) TritonX-100 in 0.01 M PBS (pH 7.4), and then reacted 1 h with FITC-conjugated-goat anti-rabbit IgG (Sigma) at 1: 400 dilution. After washing three times in precooled 0.1 M PBS (pH 7.4), samples were stained with CFW and visualized with confocal microscopy at 488 / 530 (FITC) and 405 / 435 nm (CFW). Fluorescence intensities were quantified using ImageJ software [55].

For TEM analysis, fungal cells (conidia, blastospores and hyphal bodies) were fixed, dehydrated, embedded (with Lowicryl K4M resin, Plano, Wetzlar, Germany) and sectioned (75 nm) as described previously [73]. Ultrathin sections were sequentially stained with 3% uranium acetate and lead citrate, followed by visualization for collection of TEM images. Cell wall thickness was measured using the software Image-Pro Plus (Olympus). At least 30 samples per strain were measured as described previously [50].

BbEng1 distribution in fungal cells was examined using immuno-transmission microscopy as described previously [74]. Fungal hyphal bodies isolated from insect hemolymph infected with indicated stains were used to prepare samples for immuno-transmission microscopy with anti-BbEng1 polyclonal antibody (diluted 1: 30) and anti-Rabbit IgG (Whole molecule)-Gold (Sigma-Aldrich).

### Insect bioassays and examination of intrahemocoel fungal cells

Virulence bioassays were using *G*. *mellonella* larvae and /or *L*. *migratoria manilensis* nymphs as indicated. Cuticle infection (topical bioassays) and cuticle-bypassing infection (intrahemocoel injection bioassays) using *G*. *mellonella* were initiated by spraying 1 mL aliquots of a 10^7^ conidia / mL suspension on the surfaces of larvae in a Potter Precision Laboratory Spray Tower (Burkard Manufacturing Co., Ltd., UK) or injecting 2 μL of a 5 × 10^6^ conidia / mL suspension into the larvae (*G*. *mellonlella*) or second proleg (locusts) with a microinjector (SPLab01, Baoding Shenchen Precision Pump Co., Ltd., China), respectively. Controls were treated with buffer alone (physiological saline solution). Each assay per strain included three replicates of 30–40 insects with the entire experiment repeated three times with different batches of fungal cells and insects. For *M*. *acridum* strains cuticle infection was assayed on *L*. *migratoria manilensis* nymphs by spotting 5 μL of a 10^7^ conidia / mL suspension (in liquid paraffin oil) on the pronotum of each nymph as previously described [68]. Three groups of 20 locusts were included in each assay per strain/repeated twice. Controls were treated with liquid paraffin oil only. Mortality was recorded every 12 h. The survival data were plotted as Kaplan–Meyer curves. The mean lethal time to kill 50% of targets (LT_50_) was calculated using SPSS 17.0 program.

Fungal proliferation in the hemocoel of *G*. *mellonella* (*i*.*e*., production of hyphal bodies) was examined from a subset of larvae infected through either topical infection or intrahemocoel injection as indicated. Larvae were bled at 24, 36, 48 and 60 h post-treatment and fungal development was monitored microscopically as well as via quantification of fungal cells through qPCR analysis of by amplification of *18S rRNA* sequences with primer pair 18S1 / 18S2 (S2 Table), as described previously [12,53].

### Statistical analyses

All data from three independent replicates of experiments were statistically evaluated based on one-way analysis of variance (ANOVAs) and LSD test. The Student’s test (*t*-test) was carried out to compare means between two groups or treatments. The SPSS 17.0 program were used in the analysis. Insect bioassay replicates and differences between groups were analyzed using a log rank test with GraphPad Prim 8.0.2 program.

## Supporting information

S1 Fig BbEng1 structure, and phylogenetic analysis of BbEng1 homologues and their sequence alignment. **A.** Structural diagram of BbEng1 protein. The N-terminal signal peptide and the GH16_fungal_Lam16A_glucanase (GH16) domains are indicated, in which the active sites and catalytic sites are marked with “△” and “▼”, respectively. **B.** Phylogenetic analysis of BbEng1 homologs found in insect, mammalian, and plant pathogenic fungi. **C.** Sequence alignment of BbEng1 homologs from representative species of insect, mammal and plant pathogenic fungi.
(TIF)

S2 Fig Schematic of construction of targeted gene disruption, overexpression and complementation strains. **A.** *BbEng1* locus and gene replacement vector pΔBbEng1. Homologous recombination (cross over event marked by ‘X’) resulting in a region of *BbEng1* was replaced by the bar cassette. **B.** *BbEng1* overexpression construct. *BbEng1* coding region was controlled by the *Aspergillus nidulans gpd* promoter. **C.** *BbEng1* reverse complementation vector pCB-BbEng1. **D.** PCR analysis of wild type (WT), *ΔBbEng1*, and *BbEng1* complementation (Comp) strains. Desired integration events were confirmed by PCR using primer pairs S1 / S2. **E.** RT-PCR confirmation of loss of gene expression in the *ΔBbEng1* mutant and recovery in Comp strains using *18S rRNA* as the reference gene. **F.** RT-qPCR determination of *BbEng1* transcript levels in the wild type (WT) and *BbEng1* overexpression (*BbEng1*^*OE*^) strains using primer pairs RT1 / RT2 with *18S rRNA* as a reference gene. RNAs were isolated from 1/4 SDY cultures for 48 h. **G.** Southern blot analysis of *ΔBbEng1* and Comp strains. Genomic DNAs were digested with *Hin*dIII and probed using the *bar* gene for *ΔBbEng1* and *sur* for Comp.
(TIF)

S3 Fig Colony growth, conidiation and germination of *B*. *bassiana* wild-type, *ΔBbEng1*, *BbEng1*^*OE*^ and Comp strains. **A.** Colony grown for 8 days at 26°C with a cycle consisting of 15 h of light and 9 h of darkness. Fungal strains were inoculated by dropping 2 μL of conidial suspensions (1 × 10^7^ conidia / mL) on the center of 1/4 SDAY (1:4-diluted Sabouraud dextrose agar supplemented with 1% (w/v) yeast extract), PDA, CZA and CZA replacing sucrose with glucan, trehalose, glucose, mannose, erythritol, galactose or fructose at 3% (w/v). **B.** Growth rates calculated based on colony growth in dimeters using linear regression method and software SPSS 17.0. **C.** Conidiation on CZA at indicated time. **D.** Conidial yield per cm^2^ on CZA and 1/4 SDAY at indicated time. **E.** Conidial germination on CZA and 1/4 SDAY. **F.** The mean germination time (GT_50_). All the data were present with mean and SD from triplicated independent experiments. Error bars denote st. dev. (SD) and values with different letters indicate statistically significant differences from different treatments (*P* < 0.01 in LSD test).
(TIF)

S4 Fig Dimorphic transition of *B*. *bassiana* wild-type, *ΔBbEng1*, *BbEng1*^*OE*^ and Comp strains in 1/4 SDB broth. Blastospore production (**A**) and yield (cells / mL) (**B**) at indicated time after inoculation (Scale: 10 μm). Conidia were inoculated in 30 mL 1/4 SDB broth at a final concentration of 1 × 10^5^ spores / mL and incubated at 26°C with agitation (180 rpm). All the data in (B) were repeated three times. Error bars denote st. dev. (SD) and values with different letters indicate statistically significant differences from different treatments (**P < 0.01 in *t*-test).
(TIF)

S5 Fig Cell wall structure of blastospores and conidia of indicated fungal strains. **A.** TEM micrographs of cell wall. **B.** Cell wall thickness (n = 30) (Scale: 0.2 μm). Error bars in (B) denote st. dev. (SD) and values with different letters indicate statistically significant differences from different treatments (*P* < 0.01 in LSD test).
(TIF)

S6 Fig Cell wall β-1,3-glucan and chitin contents. **A**. β-1,3-glucan and chitin contents in mycelia cultured in 1/4 SDY for 48 h. β-1,3-glucan levels were determined using an aniline blue assay as detailed in Methods section. Cell wall chitin contents were assayed with a method using HCl (6 N) hydrolyzed the SDS-extracted cell wall as detailed in Methods section. **B.** Detection of relative levels of cell wall β-1,3-glucan and chitin in blastospores and conidia (Scale: 5 μm). Cell wall β-1,3-glucan was labeled with monoclonal β-1,3-glucan specific antibody and goat anti-mouse IgG-FITC (Proteintech) after fixing cells with 3% (v/v) formaldehyde. Chitin was labeled using FITC-wheat germ agglutinin (WGA) after fixing cells with 3% (v/v) formaldehyde, as well as CFW directly stained cells. The fluorescence of the stained cells was observed using a confocal microscopy. **C.** Mean fluorescence intensities in (B) were quantified densitometrically using the ImageJ software. Error bars denote st. dev. (SD) and values with different letters indicate statistically significant differences from different treatments (*P* < 0.01 in LSD test).
(TIF)

S7 Fig Characterization of *Pichia pastoris*-expressed BbEng1. **A.** Detection of purified BbEng1 using Western blotting with anti-His Tag monoclonal antibody. M, protein ladder. **B.** BbEng1 activities to yeast glucan at different temperatures. **C.** Residue activities of BbEng1 after incubation at different temperatures as indicated for 1 h to yeast glucan. **D.** BbEng1 activities to yeast glucan at different pH values as indicated. **E.** Residue activities of BbEng1 after incubation at indicated pH values for 1 h to yeast glucan. **F.** The Kinetic curves of BbEng1 to yeast glucan at pH 6.0 and 30°C for 1 h.
(TIF)

S8 Fig TLC and HPLC analysis of BbEng1 enzyme activities against different substrates and its endo- and exo-β-1,3-glucanase activity assay. **A.** TLC analysis of the released carbohydrates from BbEng1 (2 μg) hydrolysis reactions with the indicated polysaccharides at pH 6.0 and 30°C for 1 h. G1 to G5 refer to the standard maltooligosaccharides: glucose, maltose, maltotriose, maltotetraose and maltopentaose. **B.** HPLC analysis of the released carbohydrates from BbEng1 (2 μg) hydrolysis reactions at the same conditions as those in (A). Polysaccharides include yeast glucan, barley β-glucan, pachyma, pustulan, CM-cellulose, dextran, laminaritriose, cellobiose, cuticle and colloidal chitin (1%, w/v). **C.** Assay of endo-β-1,3-glucanase and exo-β-1,3-glucanase of BbEng1 using periodate-oxidized laminarin and 4-Nitrophenyl-β-D-glucopyranoside (p-NPG) as substrates, respectively. iBbEng1 and exo-1,3-β-D-glucanase (Megazyme) (EXG) were uses as controls. The enzyme activity assay was detailed in Methods section.
(TIF)

S9 Fig Cell surface carbohydrate epitopes and β-1,3- glucan levels on blastospores and conidia. **A.** Fluorescence images of fungal cells treated with alexa Fluor 488-labeled lectins concanavalin A (ConA) and wheat germ agglutinin (WGA) and the fluorescein-labeled lectin *Galanthus nivalis* (GNL), and monoclonal β-1,3-glucan specific antibody and goat anti-mouse IgG-FITC. BL, bright light. FL, fluorescent light (Scale: 5 μm). **B.** Average fluorescence of at least 100 individual cells was measured using ImageJ software. Error bars denote st. dev. (SD) and values with different letters (a-b) indicate statistically significant differences from different treatments (*P* < 0.01 in LSD test).
(TIF)

S10 Fig Characteristics of BbEng1 homologs and their expression patterns in *Metarhizium robertsii* and *M*. *acridum*. **A.** Comparison protein structure of BbEng1 and its homologues, MrEng1 (*M*. *robertsii* Eng1) and MaEng1 (*M*. *acridum* Eng1). The N-terminal signal peptide and the GH16_fungal_Lam16A_glucanase (GH16) domain are indicated, in which the active sites and catalytic sites are marked with “△” and “▼”, respectively. **B.** Sequence alignment of BbEng1 and its homologues MrEng1 and MaEng1. The common catalytic motif in the family proteins, E-[ILV]-D-[IVAF]-[VILMF] (0,1)-E, and cysteines are labeled with “●” and “*”, respectively. **C.** Transcription patterns of *MrEng1* and *MaEng1* in *M*. *robertsii* and *M*. *acridum* different morphological cells. AHY, aerial hyphae. CO, conidia. LHY, submerged hyphae. BLS, blastospores. HB, hyphal bodies (*in vivo* blastospores). BS, the basic salt broth. Cuticle and Hemolymph, BS + 0.167 g / L silkworm cuticle or 5 mL / L hemolymph. Chitin, Glucan and Trehalose, CZB replacing sucrose with chitin, glucan or trehalose at 2%. NaCl, sorbitol, H_2_O_2_, MND, t-BHP and lactic acid, CZB containing 0.5 M NaCl,1.0 M sorbitol, 5.76 mM H_2_O_2_, 37 μM menadione or 0.78 mM tert-Butyl hydroperoxide or 2% (v/v) lactic acid. LO, low oxygen condition (with ~6% of initial O_2_ concentration). All the fungal cells cultured in different nutrients and under stress conditions for 6 h. Error bars denote st. dev. (SD) and values with different letters (a-f) indicate statistically significant differences from different treatments (*P* < 0.01 in LSD test).
(TIF)

S11 Fig Overexpression of *BbEng1* and its homologous genes, *MrEng1* and *MaEng1* in *M*. *robertsii* and *M*. *acridum*. **A.** Cassettes of gene overexpression. **B.** RT-qPCR analysis of *BbEng1*, *MrEng1* or *MaEng1* transcript levels in the overexpression strains and their wild type strains (*M*. *robertsii* and *M*. *acridum*) using primer pairs RT1 / RT2, RT3 / RT4 and RT5 / RT6 with *M*. *robertsii* or *M*. *acridum gpd* as reference genes from PDB cultures for 48 h.
(TIF)

S12 Fig Growth, conidiation and germination of *M*. *robertsii*. *M*. *acridum* and their derived overexpression strains. **A.** Colony growth on basic medium (CZA) and nutrient-rich medium (PDA) for 8 d. **B.** Fungal growth in liquid broth (PDB) for 2 d. **C.** Conidial production on CZA at indicated time after inoculation. **D.** The conidial germination and mean germination time (GT_50_) on CZA. Error bars denote st. dev. (SD) and values with different letter indicate statistically significant differences from different treatments (*P* < 0.01 in LSD test).
(TIF)

S1 Table BbEng1 enzyme activity to different polysaccharides. (DOCX)

S2 Table Primers used in this study. (DOCX)
