# Supplementary figures and images for: Insect fungal pathogens secrete a cell wall-associated glucanase that acts to help avoid recognition by the host immune system
Source: PLoS Pathog. 2023 Aug 9;19(8):e1011578. doi: 10.1371/journal.ppat.1011578 (PMC10441804; doi:10.1371/journal.ppat.1011578)

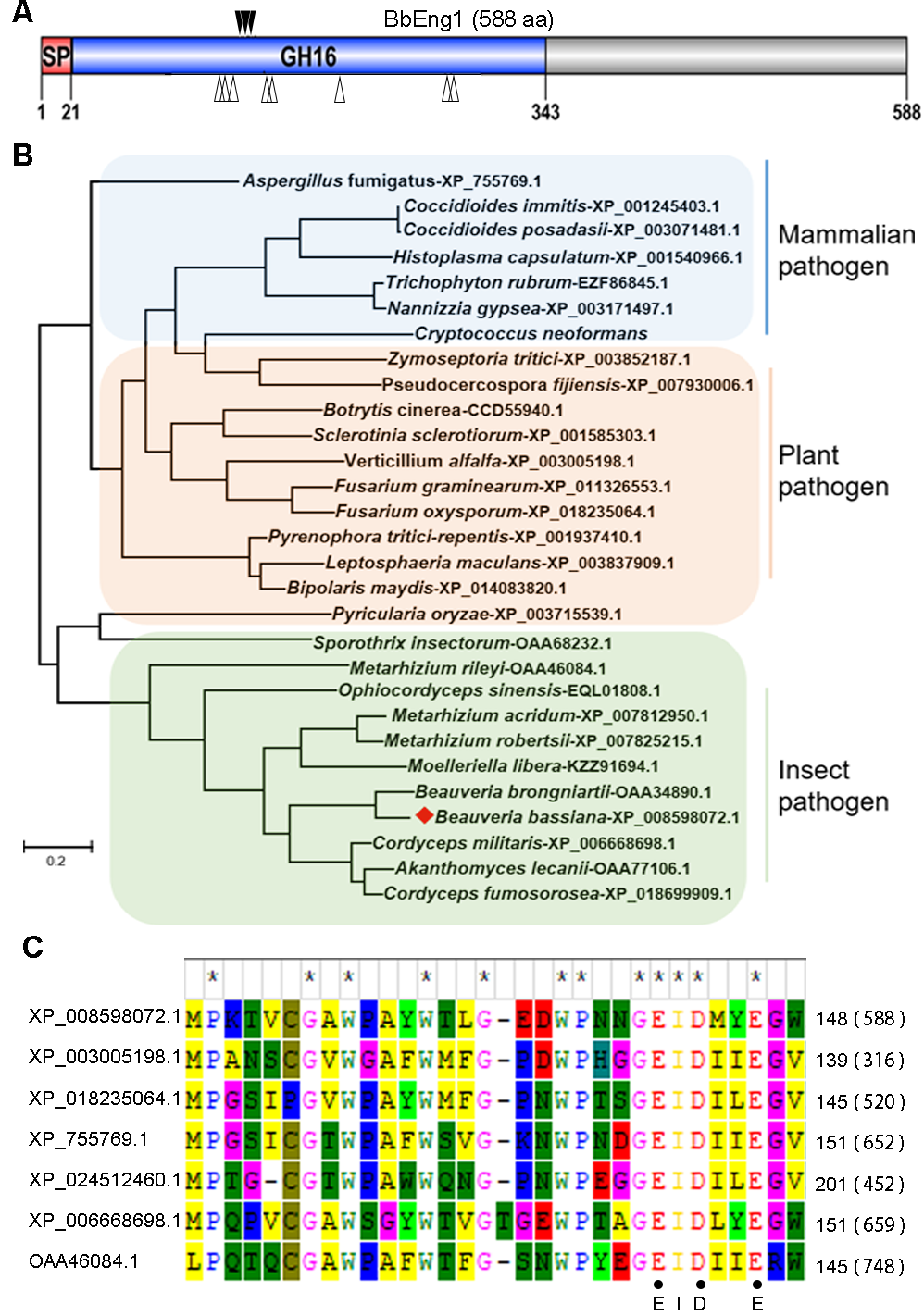

Supplement: S1 Fig — A. Structural diagram of BbEng1 protein. The N-terminal signal peptide and the GH16_fungal_Lam16A_glucanase (GH16) domains are indicated, in which the active sites and catalytic sites are marked with “△” and “▼”, respectively. B. Phylogenetic analysis of BbEng1 homologs found in insect, mammalian, and plant pathogenic fungi. C. Sequence alignment of BbEng1 homologs from representative species of insect, mammal and plant pathogenic fungi. (TIF) [file ppat.1011578.s001.tif]

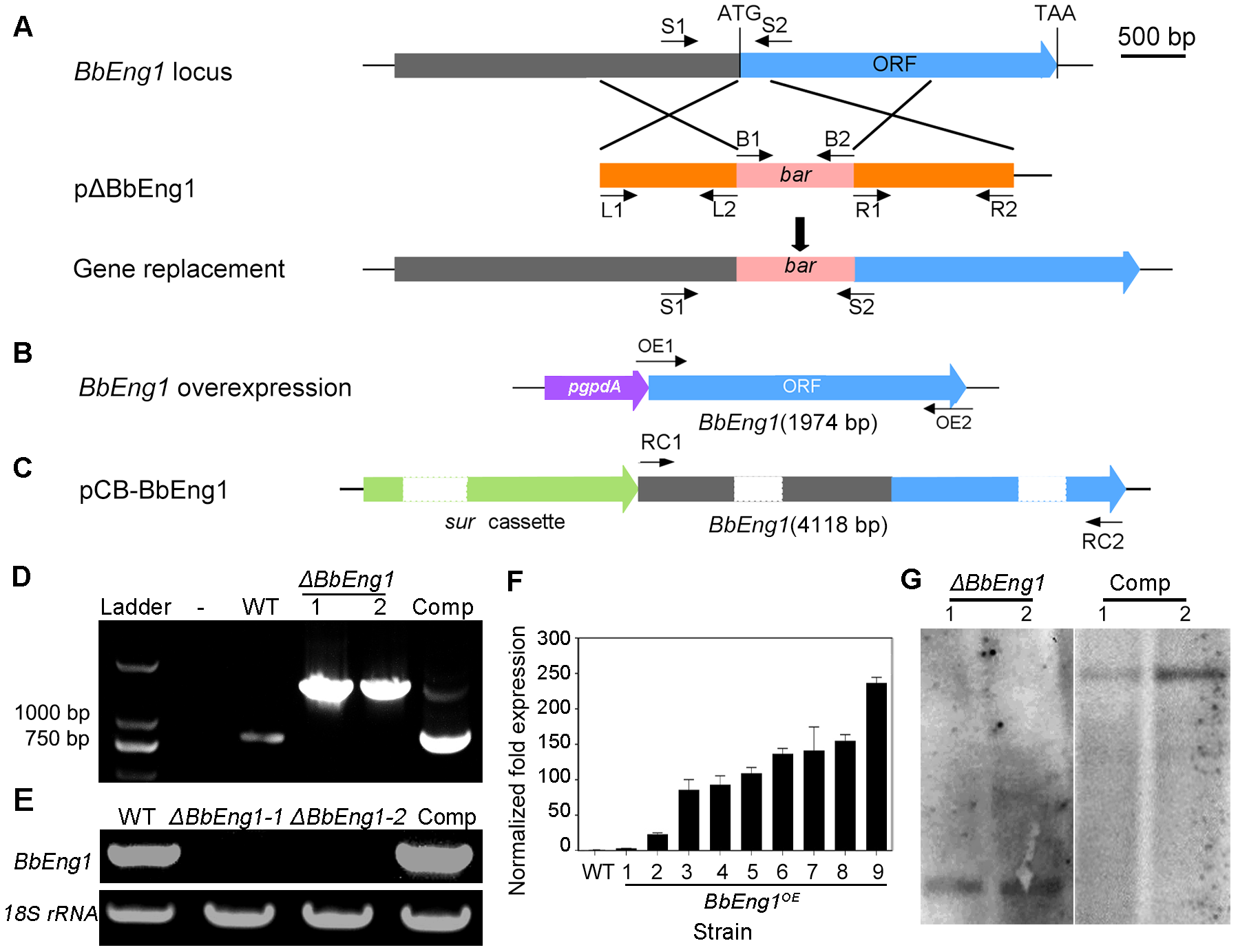

Supplement: S2 Fig — A. BbEng1 locus and gene replacement vector pΔBbEng1. Homologous recombination (cross over event marked by ‘X’) resulting in a region of BbEng1 was replaced by the bar cassette. B. BbEng1 overexpression construct. BbEng1 coding region was controlled by the Aspergillus nidulans gpd promoter. C. BbEng1 reverse complementation vector pCB-BbEng1. D. PCR analysis of wild type (WT), ΔBbEng1, and BbEng1 complementation (Comp) strains. Desired integration events were confirmed by PCR using primer pairs S1 / S2. E. RT-PCR confirmation of loss of gene expression in the ΔBbEng1 mutant and recovery in Comp strains using 18S rRNA as the reference gene. F. RT-qPCR determination of BbEng1 transcript levels in the wild type (WT) and BbEng1 overexpression (BbEng1OE) strains using primer pairs RT1 / RT2 with 18S rRNA as a reference gene. RNAs were isolated from 1/4 SDY cultures for 48 h. G. Southern blot analysis of ΔBbEng1 and Comp strains. Genomic DNAs were digested with HindIII and probed using the bar gene for ΔBbEng1 and sur for Comp. (TIF) [file ppat.1011578.s002.tif]

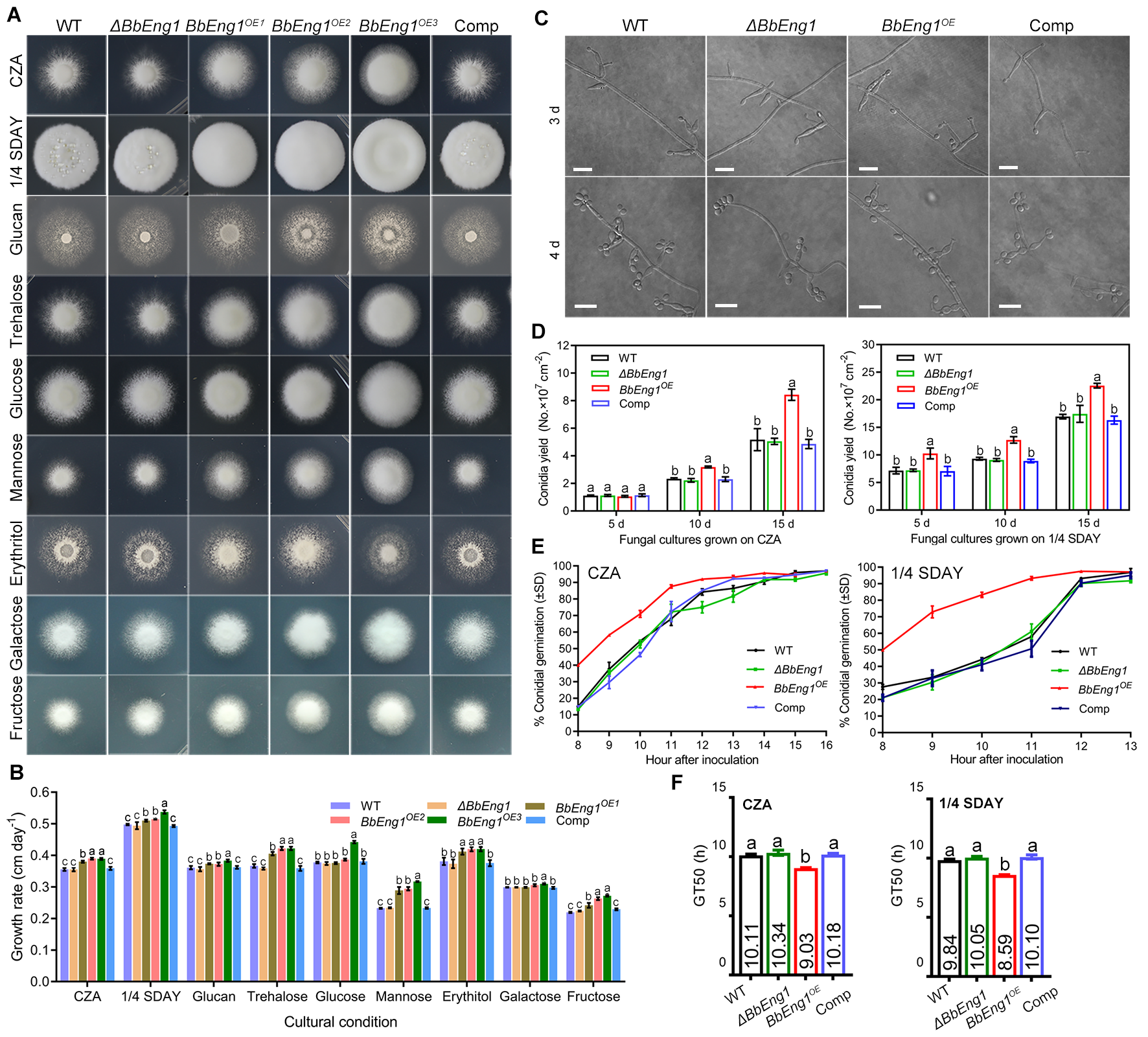

Supplement: S3 Fig — A. Colony grown for 8 days at 26°C with a cycle consisting of 15 h of light and 9 h of darkness. Fungal strains were inoculated by dropping 2 μL of conidial suspensions (1 × 10^7^ conidia / mL) on the center of 1/4 SDAY (1:4-diluted Sabouraud dextrose agar supplemented with 1% (w/v) yeast extract), PDA, CZA and CZA replacing sucrose with glucan, trehalose, glucose, mannose, erythritol, galactose or fructose at 3% (w/v). B. Growth rates calculated based on colony growth in dimeters using linear regression method and software SPSS 17.0. C. Conidiation on CZA at indicated time. D. Conidial yield per cm2 on CZA and 1/4 SDAY at indicated time. E. Conidial germination on CZA and 1/4 SDAY. F. The mean germination time (GT50). All the data were present with mean and SD from triplicated independent experiments. Error bars denote st. dev. (SD) and values with different letters indicate statistically significant differences from different treatments (P < 0.01 in LSD test). (TIF) [file ppat.1011578.s003.tif]

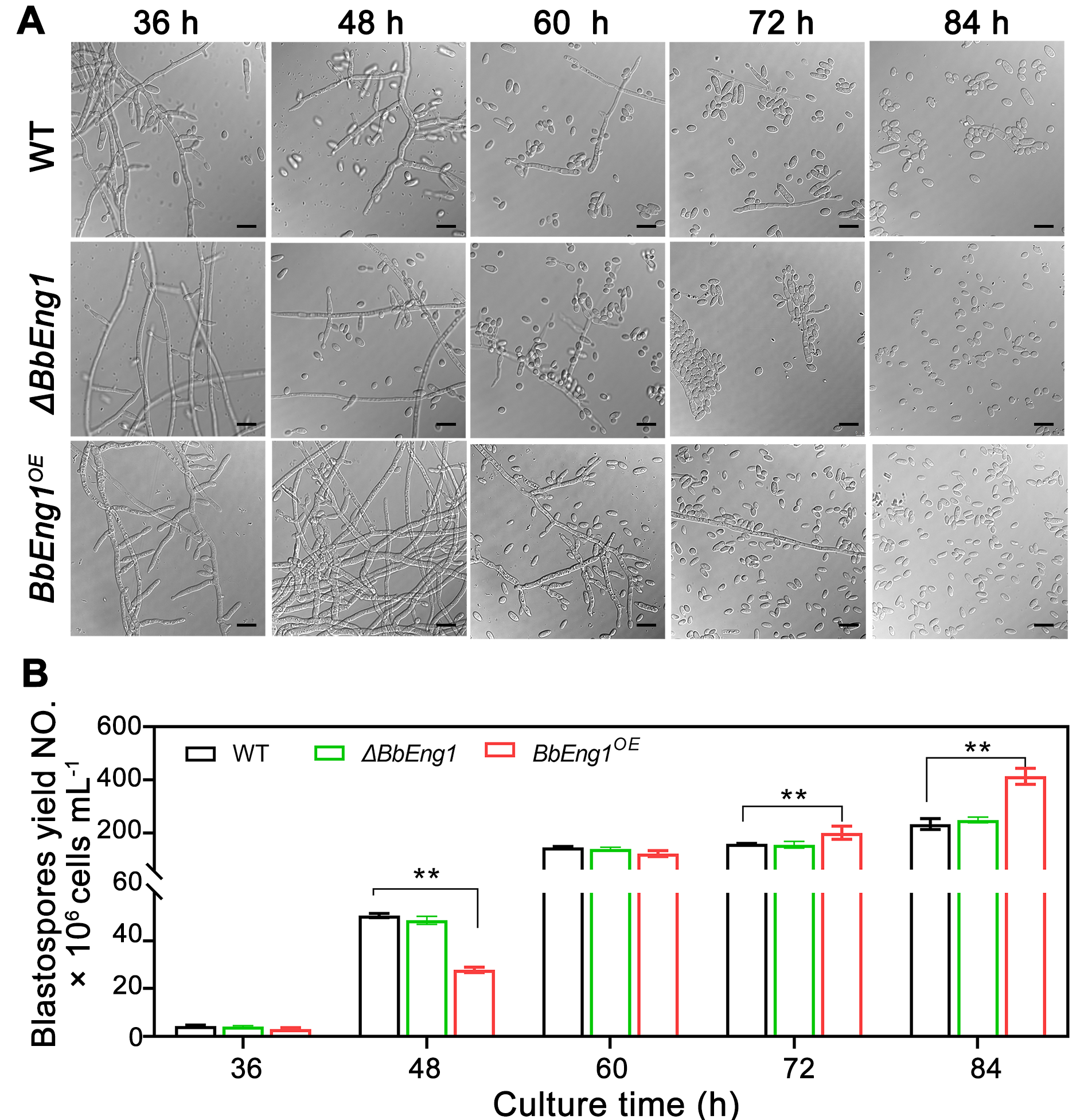

Supplement: S4 Fig — Blastospore production (A) and yield (cells / mL) (B) at indicated time after inoculation (Scale: 10 μm). Conidia were inoculated in 30 mL 1/4 SDB broth at a final concentration of 1 × 10^5^ spores / mL and incubated at 26°C with agitation (180 rpm). All the data in (B) were repeated three times. Error bars denote st. dev. (SD) and values with different letters indicate statistically significant differences from different treatments (**P < 0.01 in t-test). (TIF) [file ppat.1011578.s004.tif]

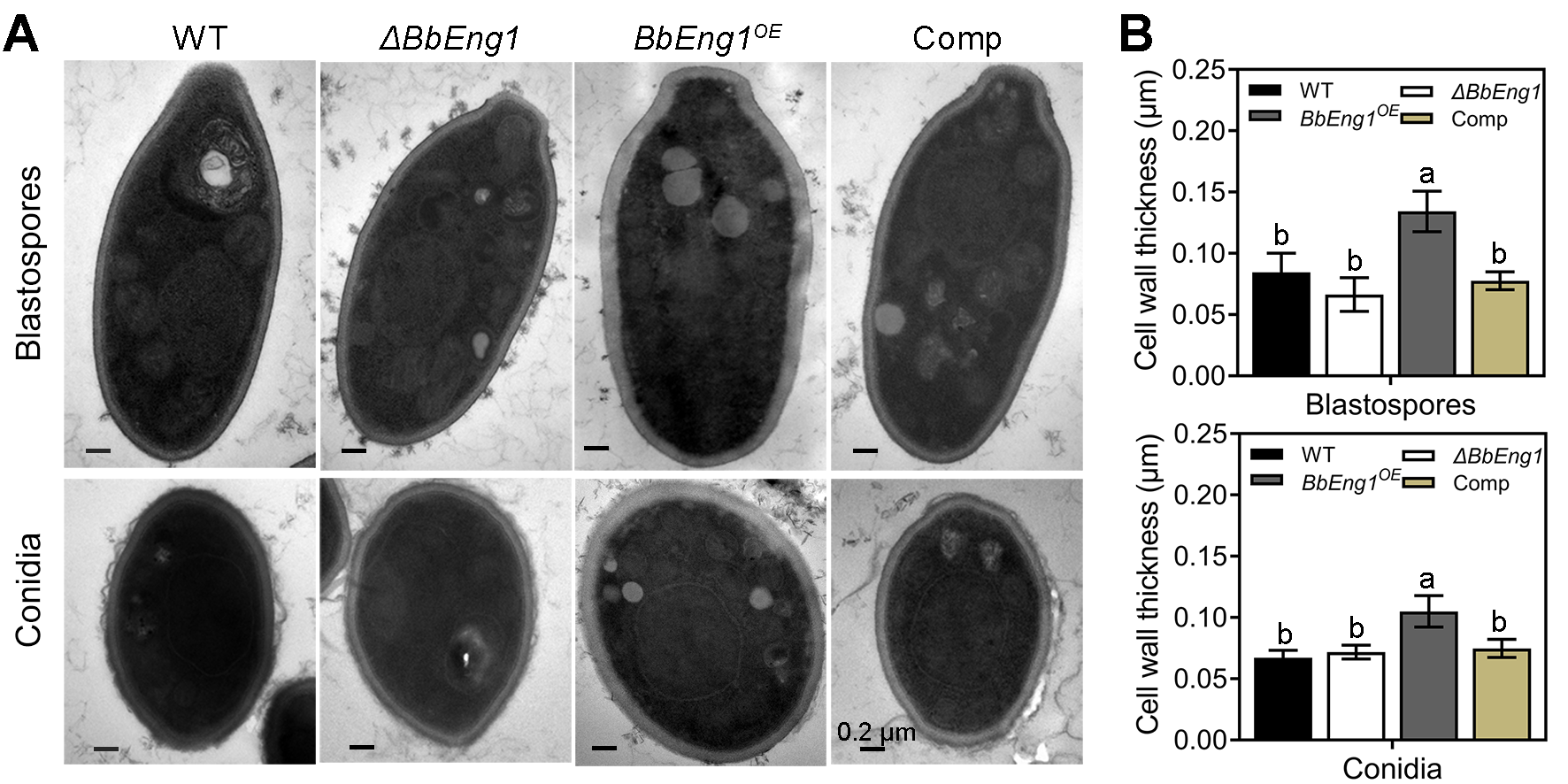

Supplement: S5 Fig — A. TEM micrographs of cell wall. B. Cell wall thickness (n = 30) (Scale: 0.2 μm). Error bars in (B) denote st. dev. (SD) and values with different letters indicate statistically significant differences from different treatments (P < 0.01 in LSD test). (TIF) [file ppat.1011578.s005.tif]

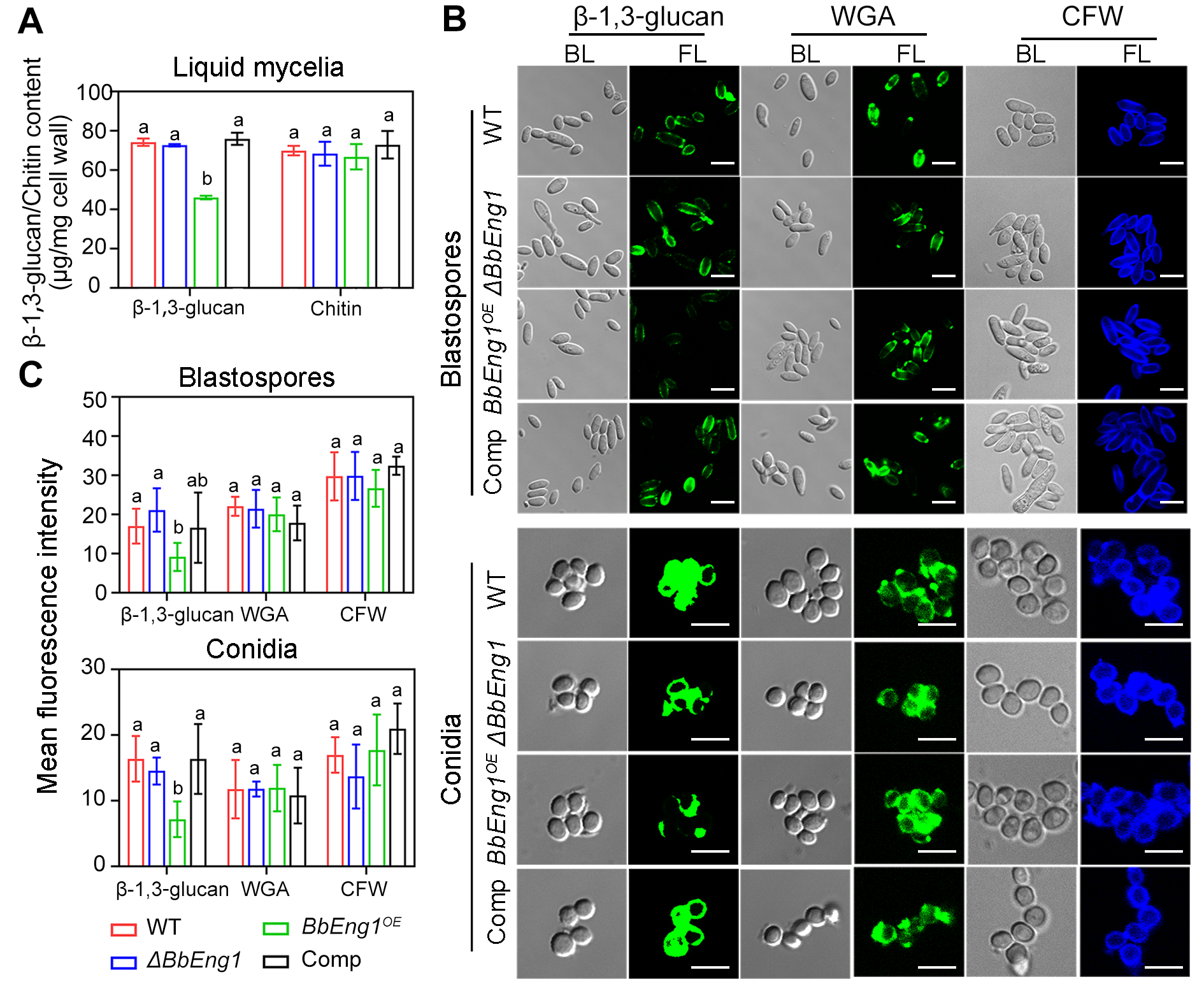

Supplement: S6 Fig — A. β-1,3-glucan and chitin contents in mycelia cultured in 1/4 SDY for 48 h. β-1,3-glucan levels were determined using an aniline blue assay as detailed in Methods section. Cell wall chitin contents were assayed with a method using HCl (6 N) hydrolyzed the SDS-extracted cell wall as detailed in Methods section. B. Detection of relative levels of cell wall β-1,3-glucan and chitin in blastospores and conidia (Scale: 5 μm). Cell wall β-1,3-glucan was labeled with monoclonal β-1,3-glucan specific antibody and goat anti-mouse IgG-FITC (Proteintech) after fixing cells with 3% (v/v) formaldehyde. Chitin was labeled using FITC-wheat germ agglutinin (WGA) after fixing cells with 3% (v/v) formaldehyde, as well as CFW directly stained cells. The fluorescence of the stained cells was observed using a confocal microscopy. C. Mean fluorescence intensities in (B) were quantified densitometrically using the ImageJ software. Error bars denote st. dev. (SD) and values with different letters indicate statistically significant differences from different treatments (P < 0.01 in LSD test). (TIF) [file ppat.1011578.s006.tif]

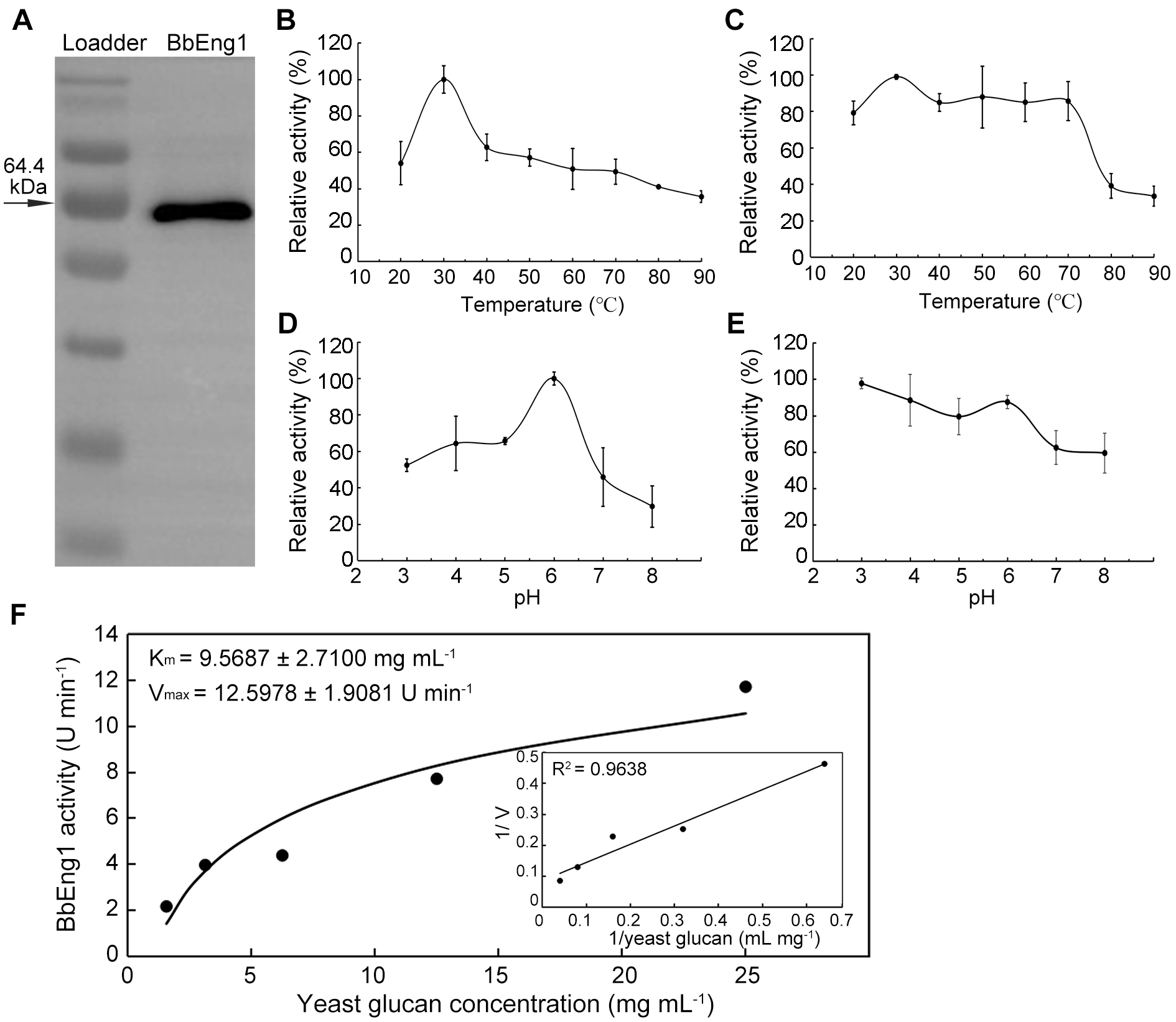

Supplement: S7 Fig — A. Detection of purified BbEng1 using Western blotting with anti-His Tag monoclonal antibody. M, protein ladder. B. BbEng1 activities to yeast glucan at different temperatures. C. Residue activities of BbEng1 after incubation at different temperatures as indicated for 1 h to yeast glucan. D. BbEng1 activities to yeast glucan at different pH values as indicated. E. Residue activities of BbEng1 after incubation at indicated pH values for 1 h to yeast glucan. F. The Kinetic curves of BbEng1 to yeast glucan at pH 6.0 and 30°C for 1 h. (TIF) [file ppat.1011578.s007.tif]

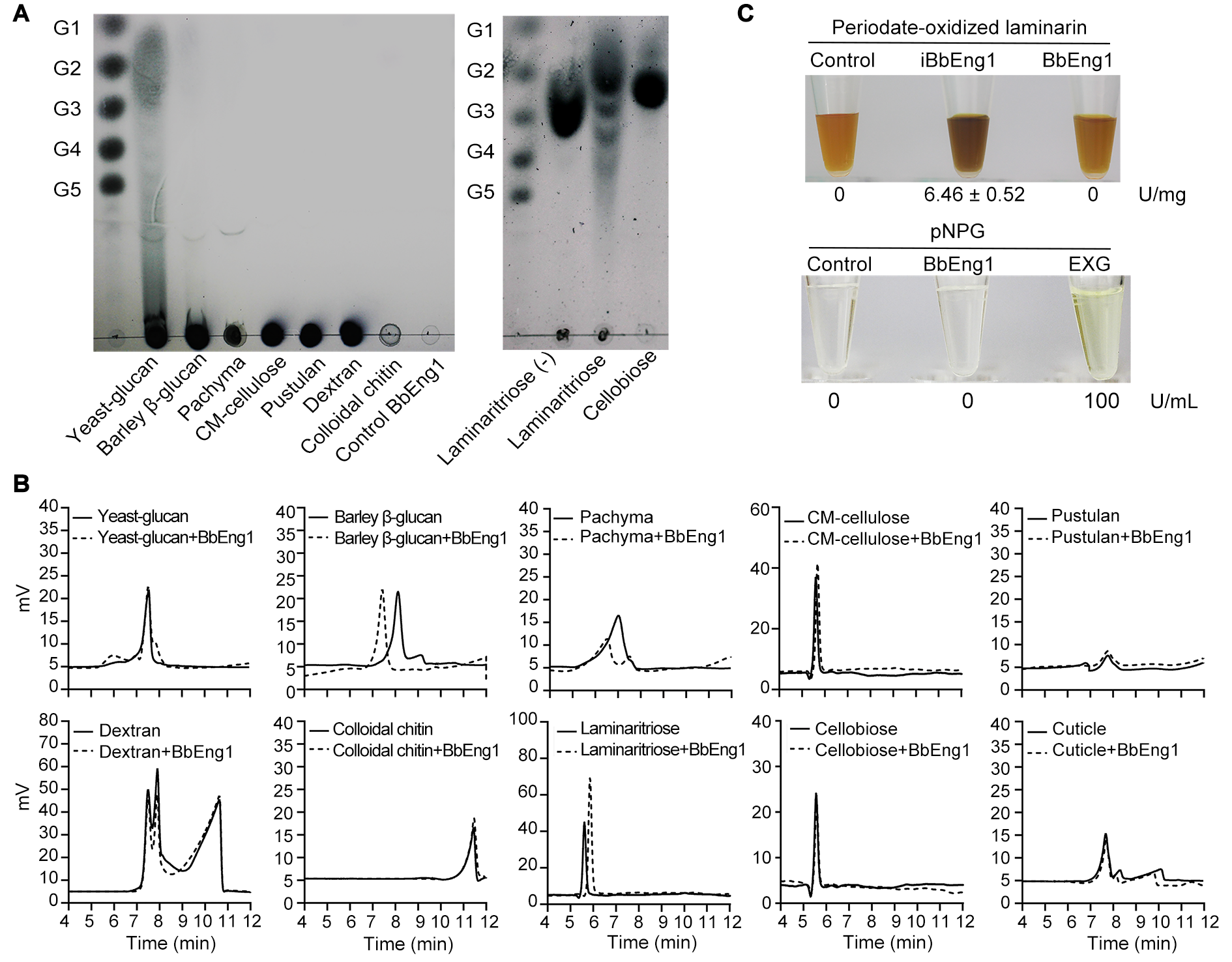

Supplement: S8 Fig — A. TLC analysis of the released carbohydrates from BbEng1 (2 μg) hydrolysis reactions with the indicated polysaccharides at pH 6.0 and 30°C for 1 h. G1 to G5 refer to the standard maltooligosaccharides: glucose, maltose, maltotriose, maltotetraose and maltopentaose. B. HPLC analysis of the released carbohydrates from BbEng1 (2 μg) hydrolysis reactions at the same conditions as those in (A). Polysaccharides include yeast glucan, barley β-glucan, pachyma, pustulan, CM-cellulose, dextran, laminaritriose, cellobiose, cuticle and colloidal chitin (1%, w/v). C. Assay of endo-β-1,3-glucanase and exo-β-1,3-glucanase of BbEng1 using periodate-oxidized laminarin and 4-Nitrophenyl-β-D-glucopyranoside (p-NPG) as substrates, respectively. iBbEng1 and exo-1,3-β-D-glucanase (Megazyme) (EXG) were uses as controls. The enzyme activity assay was detailed in Methods section. (TIF) [file ppat.1011578.s008.tif]

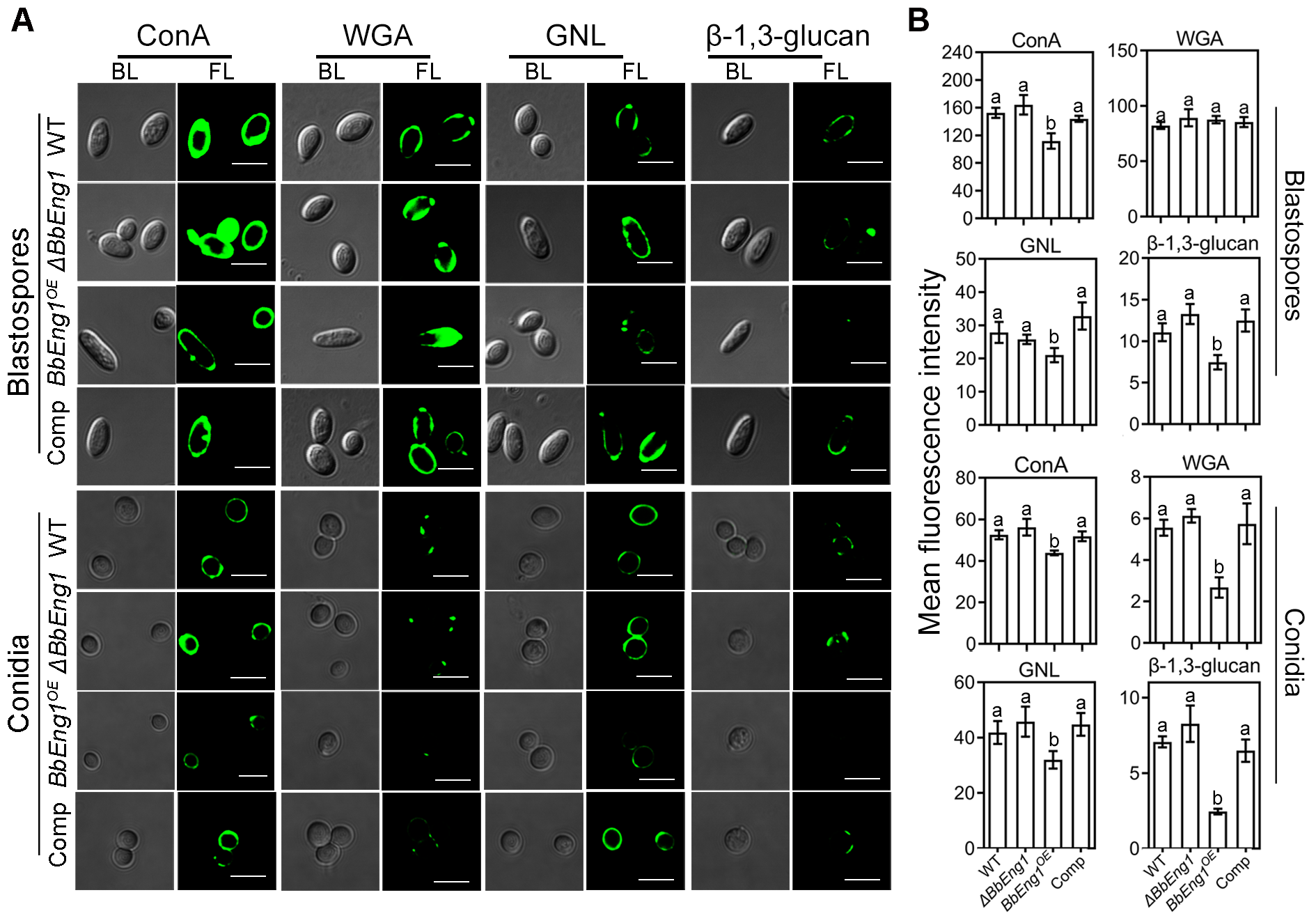

Supplement: S9 Fig — A. Fluorescence images of fungal cells treated with alexa Fluor 488-labeled lectins concanavalin A (ConA) and wheat germ agglutinin (WGA) and the fluorescein-labeled lectin Galanthus nivalis (GNL), and monoclonal β-1,3-glucan specific antibody and goat anti-mouse IgG-FITC. BL, bright light. FL, fluorescent light (Scale: 5 μm). B. Average fluorescence of at least 100 individual cells was measured using ImageJ software. Error bars denote st. dev. (SD) and values with different letters (a-b) indicate statistically significant differences from different treatments (P < 0.01 in LSD test). (TIF) [file ppat.1011578.s009.tif]

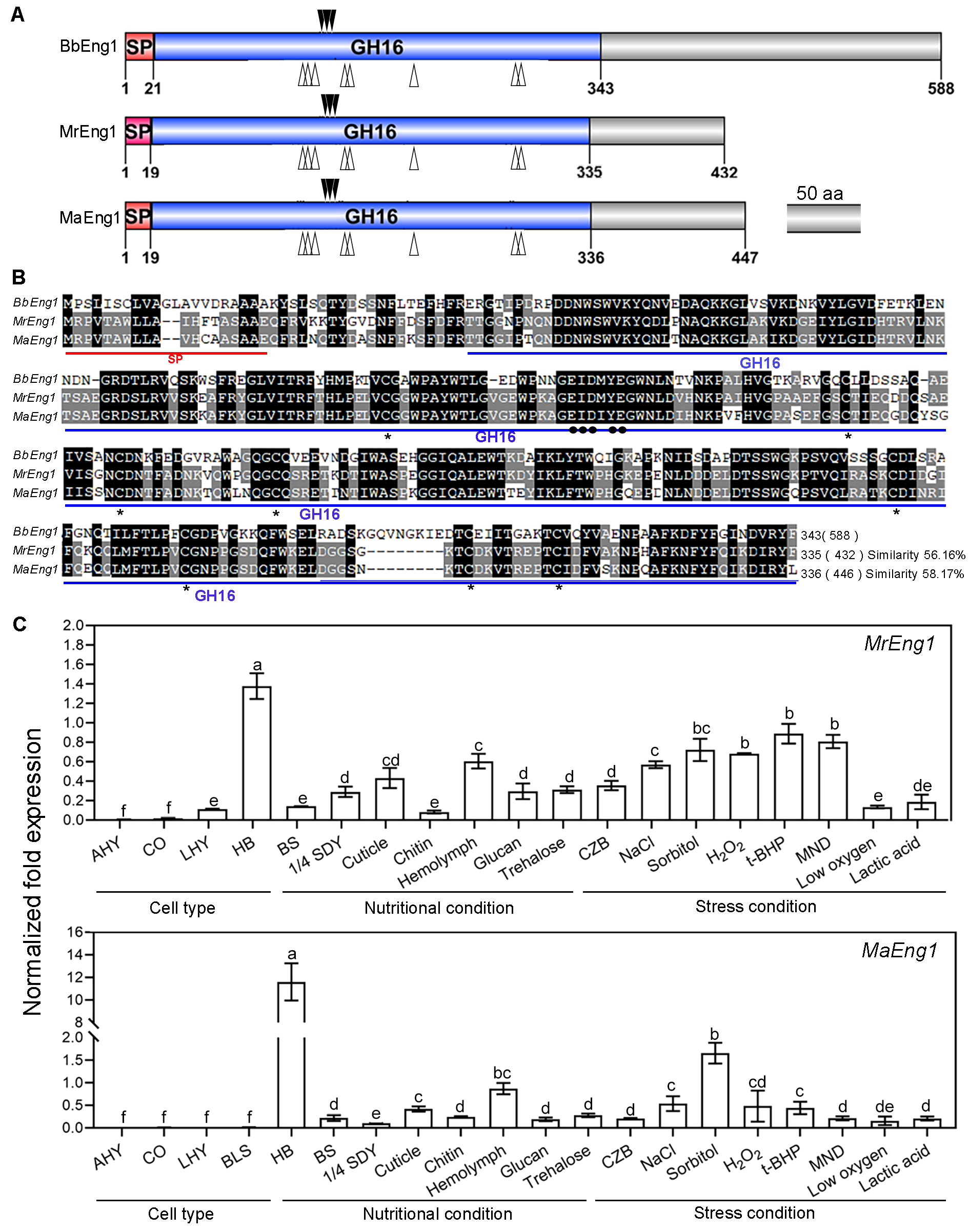

Supplement: S10 Fig — A. Comparison protein structure of BbEng1 and its homologues, MrEng1 (M. robertsii Eng1) and MaEng1 (M. acridum Eng1). The N-terminal signal peptide and the GH16_fungal_Lam16A_glucanase (GH16) domain are indicated, in which the active sites and catalytic sites are marked with “△” and “▼”, respectively. B. Sequence alignment of BbEng1 and its homologues MrEng1 and MaEng1. The common catalytic motif in the family proteins, E-[ILV]-D-[IVAF]-[VILMF] (0,1)-E, and cysteines are labeled with “●” and “*”, respectively. C. Transcription patterns of MrEng1 and MaEng1 in M. robertsii and M. acridum different morphological cells. AHY, aerial hyphae. CO, conidia. LHY, submerged hyphae. BLS, blastospores. HB, hyphal bodies (in vivo blastospores). BS, the basic salt broth. Cuticle and Hemolymph, BS + 0.167 g / L silkworm cuticle or 5 mL / L hemolymph. Chitin, Glucan and Trehalose, CZB replacing sucrose with chitin, glucan or trehalose at 2%. NaCl, sorbitol, H2O2, MND, t-BHP and lactic acid, CZB containing 0.5 M NaCl,1.0 M sorbitol, 5.76 mM H2O2, 37 μM menadione or 0.78 mM tert-Butyl hydroperoxide or 2% (v/v) lactic acid. LO, low oxygen condition (with ~6% of initial O2 concentration). All the fungal cells cultured in different nutrients and under stress conditions for 6 h. Error bars denote st. dev. (SD) and values with different letters (a-f) indicate statistically significant differences from different treatments (P < 0.01 in LSD test). (TIF) [file ppat.1011578.s010.tif]

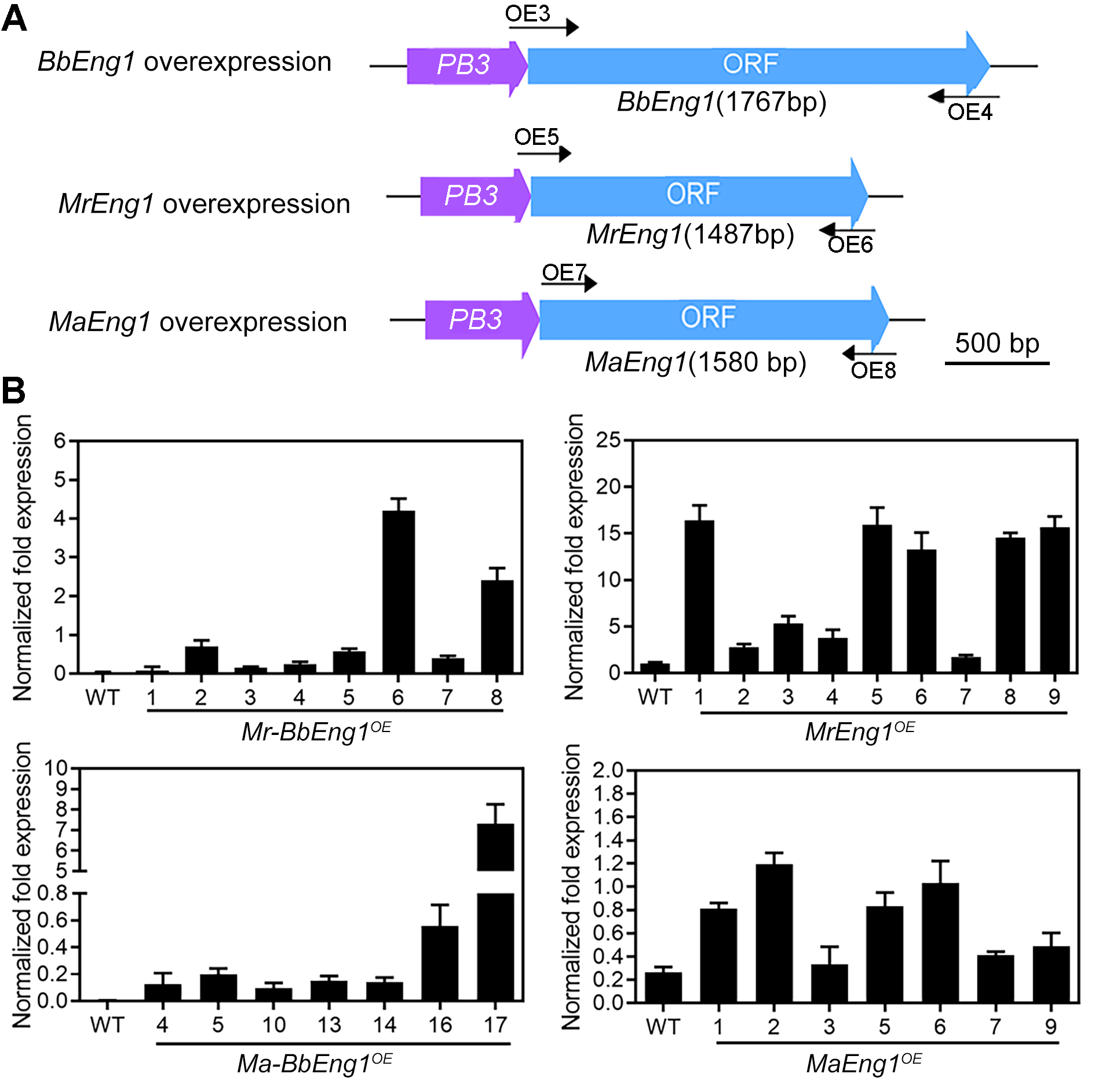

Supplement: S11 Fig — A. Cassettes of gene overexpression. B. RT-qPCR analysis of BbEng1, MrEng1 or MaEng1 transcript levels in the overexpression strains and their wild type strains (M. robertsii and M. acridum) using primer pairs RT1 / RT2, RT3 / RT4 and RT5 / RT6 with M. robertsii or M. acridum gpd as reference genes from PDB cultures for 48 h. (TIF) [file ppat.1011578.s011.tif]

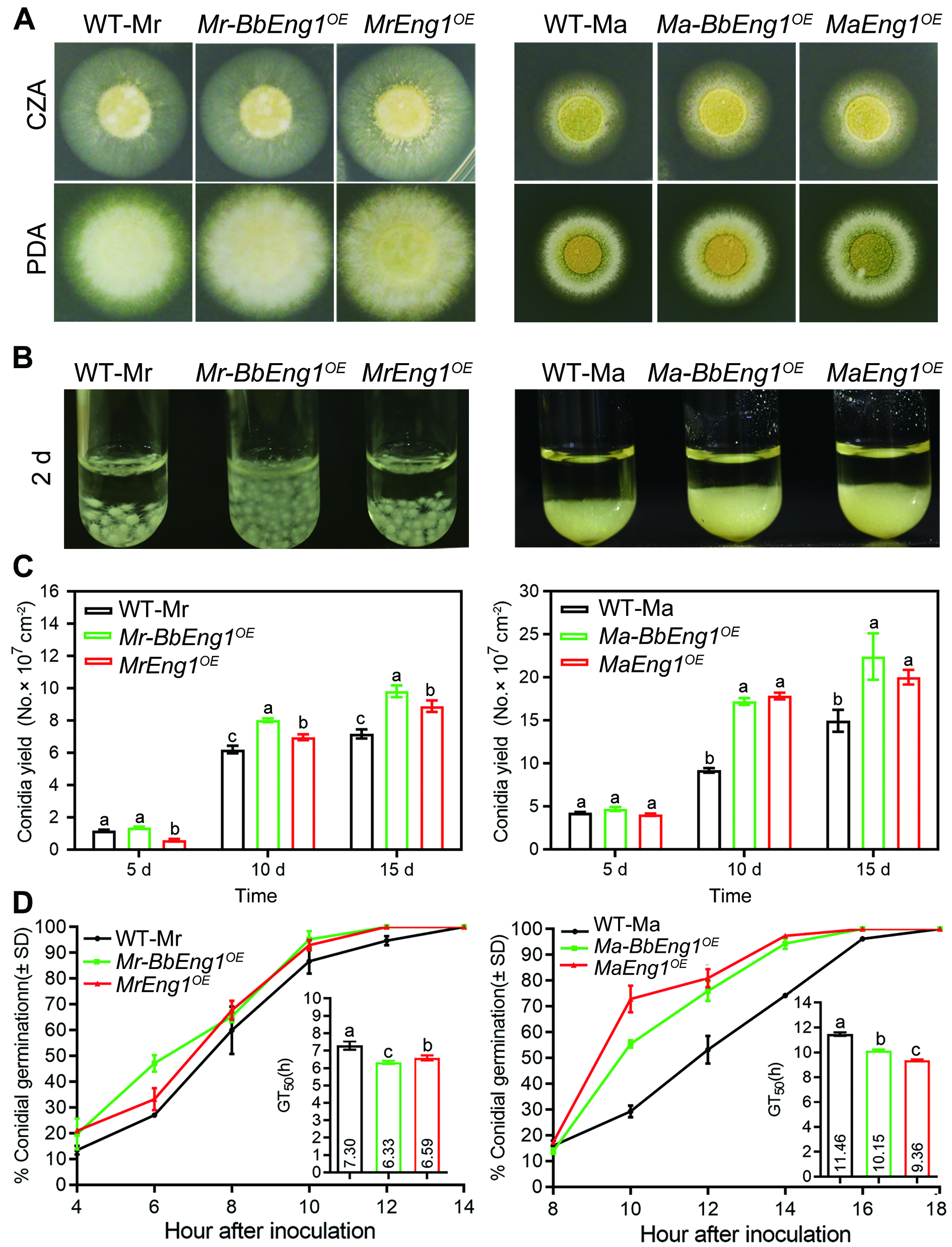

Supplement: S12 Fig — A. Colony growth on basic medium (CZA) and nutrient-rich medium (PDA) for 8 d. B. Fungal growth in liquid broth (PDB) for 2 d. C. Conidial production on CZA at indicated time after inoculation. D. The conidial germination and mean germination time (GT50) on CZA. Error bars denote st. dev. (SD) and values with different letter indicate statistically significant differences from different treatments (P < 0.01 in LSD test). (TIF) [file ppat.1011578.s012.tif]
