# Supplementary material for: Insect fungal pathogens secrete a cell wall-associated glucanase that acts to help avoid recognition by the host immune system
Source: PLoS Pathog. 2023 Aug 9;19(8):e1011578. doi: 10.1371/journal.ppat.1011578 (PMC10441804; doi:10.1371/journal.ppat.1011578)
Supplement: S1 Table — (DOCX) [file ppat.1011578.s013.docx]

**S1 Table. Degradation activity of BbEng1 to different substrates polysaccharides**

| **Substrate** | **Glucosidic bond** | **Substrate specificity (U/ mg)*** |
| --- | --- | --- |
| barley β-glucan | β-1,3/1,4-(glucose) | 43.71 ± 1.35 |
| yeast glucan | β-1,3/1,6-(glucose) | 56.60 ± 2.41 |
| pachyma | β-1,3-(glucose) | 36.47 ± 1.35 |
| CM-cellulose | β-1,4-(glucose) | 0 |
| dextran | α-1,3/1,6-(glucose) | 0 |
| pustulan | β-1,6-(glucose) | 0 |
| colloidal chitin | β-1,4-(N-Acetyl-D-glucosamine) | 0 |
| laminaritetraose | β-1,3-(glucose) | 99.86 ± 11.48 |
| cellobiose | β-1,4-(glucose) | 0 |

* BbEng1 activities against substrates were assayed with the DNS method except laminaritetraose and cellobiose which were assayed with a glucose oxidase method. One unit of glucanase activity is defined as the amount of enzyme that releases 1 μmol of reducing sugar (glucose) per minute per μg of protein under the tested conditions.
