# Supplementary material for: Insect fungal pathogens secrete a cell wall-associated glucanase that acts to help avoid recognition by the host immune system
Source: PLoS Pathog. 2023 Aug 9;19(8):e1011578. doi: 10.1371/journal.ppat.1011578 (PMC10441804; doi:10.1371/journal.ppat.1011578)
Supplement: S2 Table — (DOCX) [file ppat.1011578.s014.docx]

**S2 Table. Primers used in this study**

| **Primer** | **Sequence** | **Remarks** |
| --- | --- | --- |
| Constructing *GFP* fusion vectors and screening *B. bassiana* transformants | | |
| C1 | ACGACGGCCAGTGCCAAGCTTTCTTGCCGACCCTGTTCTTC | cloning *BbEng1* (BBA_04753) promoter |
| C2 | GCCCTTGCTCACCATGATATCGACGGTAAAGACTGTATGGCG |  |
| C3 | CGGTATTTCACACCGCATATGTAGCTGATGCTCTCCGCGTC | cloning *BbEng1* containing promoter sequence |
| C4 | GCCCTTGCTCACCATGATATCGGCACGGCAGATTTGGTTGG |  |
| RT1 | CTACAAGCCAGAGTCGTCCTC | confirmation of transformant with PCR |
| GFP2 | TCTCGTTGGGGTCTTTGCTC |  |
| RT-PCR and RT-qPCR analysis of *B. bassiana* genes | | |
| RT2 | AGGTGCCCTGCTGGAT | RT-PCR and qRT-PCR analysis of *BbEng1* |
| 18S1 | ACGGGTAACGGAGGGTTAGG | *18S rRNA* (Gen-Bank ID:EU334679) |
| 18S2 | AGTACACGCGGTGAGGCGGA |  |
| Constructing *BbEng1* disruption vector p∆BbEng1 and screening disruption strain | | |
| L1 | ACATGATTACGAATTCGGGTGTCTGTTTTGTGTGCG | cloning end of *BbEng1* |
| L2 | CAATGTCATCTTCTGTCGACCTGTATGGCGTGTGAGGCAA |  |
| R1 | TGCCCGTCACCGAGATCTAAGGTACAATTCCGGATCGGCCA | cloning 3'-end of *BbEng1* |
| R2 | CAACACTAGTGGATCCGGTTCTCGGCAACGTACTGA |  |
| B1 | TTGCCTCACACGCCATACAGGTCGACAGAAGATGACATTG | cloning *bar* cassette |
| B2 | TGGCCGATCCGGAATTGTACCTTAGATCTCGGTGACGGGCA |  |
| B3 | ACCTTCTTAAGTTCGCCCTT | cloning bar fragment for Southern blotting |
| B4 | GTAGAGCGTGGAGCCCAGT |  |
| S3 | TGGTAGCACTCTCGCAGTTG | confirmation of transformant with PCR |
| S4 | CTCAAAGTCCACGCCCAGAT |  |
| Constructing *BbEng1* reverse complement vector and screening and screening disruption strain | | |
| RC1 | TGCTCTCACGTCGACGGATCCATTGGCAGAGGTGTCTCCAC | cloning *BbEng1* containing promoter sequence |
| RC2 | TGCCTGCAGGTCGACTCTAGATTAGGCACGGCAGATTTGGT |  |
| Sur1 | AGTGTGCTGAGGAGGGCTAT | Cloning sur fragment for Southern blotting |
| Sur2 | ACACGGTCATCGAAGCGGCCA |  |
| Cloning *BbEng1* sequence used for construction of overexpression vector PBAGPE1-BbEng1 | | |
| OE1 | CGCGGATCCATGCCGTCACTCATTTCGTG | cloning ORF of *BbEng1* |
| OE2 | GGAATTCGGCACGGCAGATTTGGTTGG |  |
| S3 | TGAGAAGGTTTTGGGACGCT | confirmation of transformant with PCR |
| S4 | CGACCATTTGGATTGGACGC |  |
| Constructing pPIC9K vectors and screening yeast transformants | | |
| P1 | CCGGAATTCATGCATCATCACCATCACCATAAGTATTCGCTGTCCCAAA | expression of *BbEng1* without signal peptide |
| P2 | AAGGAAAAAAGCGGCCGCTTAATGGTGATGGTGATGATGGGCACGGCAGATTTGGTTG |  |
| RT-qPCR analysis antimicrobial peptide proteins (AMPs) genes of the insect *Galleria mellonella* | | |
| β-actin1 | ATCTGGCATCACACCTTCTACAACG | *β-actin* (XM_026904349) |
| β-actin2 | GACATACATAGCCGGGGAGTTGAAG |  |
| Cec1 | ATTTGCCTGCATCGTAGCG | *Cec* (XM_026898304.2) |
| Cec2 | CTTGTACTGCTGGACCAGCTTTT |  |
| Gal-P1 | GTGGGGTGCGACGAATTACA | *Gal-P* (AY528421) |
| Gal-P2 | CAAGAAGCTGCCGCAATGAC |  |
| Mor-A1 | TGCCCGTTGGTGCCATAAAA | *Mor-A* (EF564370.1) |
| Mor-A2 | GGCTGTATACTTCGTGCGCT |  |
| Mor-B1 | TGGTAAAGCTCTGCGTGGAA | *Mor-B* (EF564366.1) |
| Mor-B2 | TCTTTTTCGGTTTGAACTGGCT |  |
| AP2-1 | GTGCAAAATGCCTTTGACTCG | *AP2* (JQ862476.1) |
| AP2-2 | TTGGCGCTTCTTTCTTCTCTGT |  |
| AAP1 | TCCGTTTTGTTGTTGGTCTGC | *AAP* (DI105103.1) |
| AAP2 | CACACGCACCTCCCTATCAG |  |
| Prp1-1 | CTACCGCATCCATGGTCTCC | *PP1*(FJ494919.1) |
| Prp1-2 | CTTTGCCACGGTTGTGTACG |  |
| Glo1 | CGTTAGCAAGCAGATGCACG | *Glo*(AF394588.1) |
| Glo2 | ATTTCGCCATTTCTGCCGAC |  |
| RT-qPCR analysis the Toll pathway genes of the insect *Galleria mellonella* | | |
| βGRP1-1 | AGAATGCCGACTGGTGACTG | βGRP1 (AM265582.1) |
| βGRP1-2 | GGATATGCCATCAGGCCTCC |  |
| Spätzle1 | TCTGGGCCAACAACACTAGG | *Spätzle* (XM_031908808) |
| Spätzle2 | ACCAGTCAGCGAAGATACCG |  |
| Dorsal1 | TAAAGCGCGATCGTACGGAG | *Dorsal* (XM_031907032) |
| Dorsal-2 | CCGTGAAGGGATATGTGCGT |  |
| PGRP1 | TCGGTGCGCATACTAGAGGT | peptidoglycan recognition protein (AAN15786.1) |
| PGRP2 | TCTCTGTAGCGATGAGCTGC |  |
| RT-qPCR analysis the Serine cascade pathway genes of the insect *Galleria mellonella* | | |
| prPO1 | CCGTAACCTCACCGACTTCC | *PrPO* (AF336289) |
| prPO2 | TATTGGCGTTGTGTCCCCTC |  |
| PAF2-1 | GGACGTTAACAACGCTTCGG | *PAF* (AF394589) |
| PAF2-2 | GTTCGACGCTCTCCTGACAT |  |
| Constructing overexpression *BbEng1* vector and screening *Metarhizium* transformants | | |
| OE3 | CCCTTTTAATCAATAACAGGATCCATGCCGTCACTCATTTCGTG | cloning ORF of *BbEng1* cDNA |
| OE4 | TCGACGGTATCGATAAGCTTGATATCTTAGGCACGGCAGATTTGGT |  |
| S5 | AATCCGTGCCCACGACTACAA-3 | confirmation of transformant with PCR |
| S6 | CGACCATTTGGATTGGACGC |  |
| Constructing overexpression *MrEng1* vector and screening *M. robertsii* transformants | | |
| Mrgpd1 | GACTGCCCGCATTGAGAAG | *Gpd* (MAA_07675)，reference gene |
| Mrgpd2 | GCTTGACAAAGTTCTTGTTG |  |
| RT3 | CCACATCCCTGACGACAACA | *MaEng1* (MAA_09026) |
| RT4 | CGCACCATGTCTATGCTTGC |  |
| OE5 | CCCTTTTAATCAATAACAGGATCCATGCGCCCCGTAACCGCTTG | cloning ORF of *MaEng1* |
| OE6 | TCGACGGTATCGATAAGCTTGATATCTTAGATGTGTCGCAAACCAT |  |
| S7 | AATCCGTGCCCACGACTACAA | confirmation of transformant with PCR |
| S8 | CGCACCATGTCTATGCTTGC |  |
| Constructing overexpression *MaEng1* vector and screening *M. acridum* transformants | | |
| Magpd1 | GACTGCCCGCATTGAGAAG | *Gpd* (MAC_09584)，reference gene |
| Magpd2 | GCTTGACAAAGTTCTTGTTG |  |
| RT5 | AGGCTCATCAAGCCAACGAA | RT-PCR and qRT-PCR analysis of *MrEng1* (MAC_06610) |
| RT6 | TGGGTATCCTTTTGGCCGTC |  |
| OE7 | CCCTTTTAATCAATAACAGGATCCATGCGCCCTGTAACCGCTTG | cloning ORF of MrEng1 |
| OE8 | TCGACGGTATCGATAAGCTTGATATCTTAGATGTGTCGCGCACCAT |  |
| S9 | AATCCGTGCCCACGACTACAA | confirmation of transformant with PCR |
| S10 | TGGGTATCCTTTTGGCCGTC |  |
